# Development of a high-throughput screening platform for *C. difficile* toxin synthesis inhibitors unveils meclizine as an antivirulence agent

**DOI:** 10.1128/aac.00960-25

**Published:** 2025-12-17

**Authors:** Ravi K. R. Marreddy, Nghi Nguyen, Chetna Dureja, Ann Marie McKelvey, Reid Powell, Abiola O. Olaitan, Clifford Stephan, Julian G. Hurdle

**Affiliations:** 1 Center for Infectious and Inflammatory Diseases, Institute of Biosciences and Technology, Texas A&M Health Science Center 26514 , Houston, Texas, USA; 2 Center for Translational Cancer Research, Institute of Biosciences and Technology, Texas A&M Health Science Center 26514 , Houston, Texas, USA; The Peter Doherty Institute for Infection and Immunity, Melbourne, Victoria, Australia

**Keywords:** *Clostridioides difficile*, assay development, small-molecule inhibitor screening, toxins, luciferase

## Abstract

*Clostridioides difficile,* a leading cause of hospital-acquired diarrhea, exerts its virulence through two co-regulated toxins, TcdA and TcdB. Despite their pivotal roles, the discovery of inhibitors targeting their biosynthesis is underexplored. Here, we present a high-throughput screening (HTS) platform designed to identify toxin synthesis inhibitors (TSIs) that minimally impact bacterial growth. The primary screen utilized a *C. difficile* reporter strain expressing secreted Nano-luciferase (secNluc) under the *tcdA* promoter, whereby inhibition of secNluc production indicates toxin biosynthesis inhibition. Screening the Prestwick Chemical Library at 10 and 100 µM identified several compounds that reduced secNluc activity. Through counter-screening, we eliminated compounds that caused spectral interference. Orthogonal dose-response assays assessing the effectiveness of inhibiting toxin production without affecting growth identified meclizine, an antihistamine, as the primary antivirulence candidate. Meclizine was confirmed as a TSI by showing that it reduced TcdA and TcdB protein levels, the cytopathic potential of cultures, and *tcdA* and *tcdB* transcription as determined by ELISA, cell-rounding assays, and RT-qPCR, respectively. Meclizine significantly altered central carbon metabolism in *C. difficile*, upregulating carbohydrate transport systems and the conversion of lactate to pyruvate, while downregulating glycolytic genes. These changes were associated with intracellular accumulation of glucose and pyruvate, metabolites known to negatively impact toxin production. Taken together, our findings underscore the utility of the above HTS platform to identify anti-*C*. *difficile* TSIs, which can serve as molecular and cellular probes, as well as chemical starting points for developing novel therapeutics for *C. difficile* infection.

## INTRODUCTION

*Clostridioides difficile*, a gram-positive anaerobe, is a leading cause of hospital-acquired diarrhea, responsible for 20,500 deaths from 462,100 infections in 2017 (1, 2). The primary risk factor for *C. difficile* infection (CDI) is broad-spectrum antimicrobials, which disrupt the gut microbiota, leading to dysbiosis that facilitates colonization and disease development by *C. difficile*. Vancomycin and metronidazole, traditionally first-line treatments, also exacerbate dysbiosis and contribute to recurrent CDI, which occurs in 20% or more of patients (3, 4). This emphasizes the need for narrow-spectrum anti-*C*. *difficile* agents that preserve the gut microbiota. Fidaxomicin exemplifies this approach, as it has a lower recurrence rate than vancomycin (5, 6). Although other narrow-spectrum antimicrobials and non-antimicrobials are under development, high attrition rates for CDI drugs in development highlight the need for new agents to sustain the pipeline for CDI therapeutics (7, 8). In this study, we focused on developing a discovery platform for non-antimicrobials that inhibit cellular production of *C. difficile* toxins.

*C. difficile* produces two main virulence factors, toxin A (TcdA) and toxin B (TcdB), which are glycosyltransferase toxins targeting the Rho family of GTPases (Rho, Rac, and Cdc42) (9). TcdA and TcdB disrupt cells’ actin cytoskeleton, leading to epithelial damage and inflammation that is visualized as cell rounding in tissue cultures (9). Current antivirulence strategies against *C. difficile* primarily focus on blocking the actions of TcdA and TcdB, with the leading example being the monoclonal antibody bezlotoxumab, which targets TcdB (10–12). In contrast, there have been limited efforts to discover inhibitors that block the cellular synthesis of TcdA and TcdB (13). This is in stark contrast to other major hospital pathogens, such as *Escherichia coli*, *Staphylococcus aureus*, and *Pseudomonas aeruginosa*, which have received substantial research attention aimed at discovering antivirulence agents (14–16). TcdA and TcdB are co-regulated and produced during the late-log and stationary phases of growth, with their biosynthesis attenuated under specific metabolic conditions (17). Consequently, targeting the biosynthesis of either toxin could effectively inhibit both. For example, the transcription of *tcdA* and *tcdB* is repressed by glucose through carbon catabolite repression, mediated by the catabolite control protein A (CcpA) (18–20), by CodY, a nutritional sensor that responds to elevated levels of GTP or branch-chained amino acids (21), or by pyruvate via a mechanism that is not clearly defined (22).

**Fig 1:**
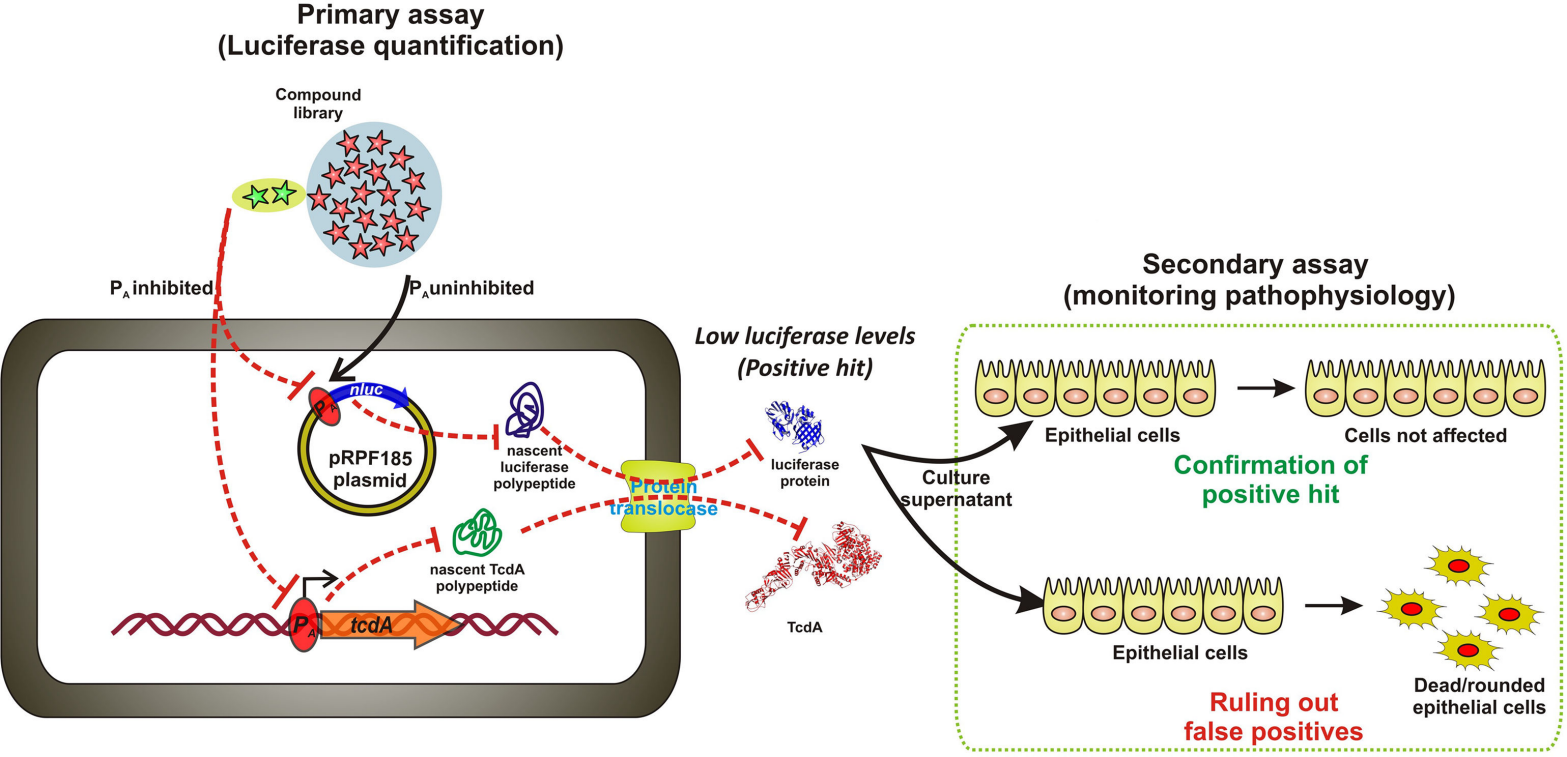
Schematic of the screening strategy for inhibitors of *C. difficile* toxin production. In the primary assay, the *tcdA* promoter (PtcdA) drives the expression of a secreted NanoLuc (secNluc) reporter in strain R20291[PtcdA::secNluc]. Because NanoLuc expression correlates with tcdA transcription, compounds that reduce luminescence are predicted to inhibit the production of the co-regulated toxins TcdA and TcdB. In the secondary assay, toxin activity in culture supernatants is assessed by epithelial cell rounding, where unaffected cells indicate true toxin inhibition and rounded or dead cells indicate false positives. (ELISA-based detection of TcdA and TcdB was also used for hit confirmation but is not shown).

The lack of systemic efforts to identify inhibitors affecting these or other pathways may be attributed to several factors, including the complexity of working with anaerobic bacteria or their proteins in high-throughput screening (HTS) platforms, as well as limited insights into the druggability of target proteins mediating toxin biosynthesis (17, 23). To overcome the first of these challenges, we developed an HTS assay performed in 384-well plates using a *C. difficile* reporter strain engineered to express secreted NanoLuc Luciferase (secNluc) from the *tcdA* promoter, providing a quantifiable proxy metric for toxin production (Fig. 1). Here, we describe the development and application of this HTS platform, which facilitated the discovery of the antihistamine meclizine as an inhibitor of TcdA and TcdB biosynthesis. Initial mechanistic studies suggest that meclizine achieves this by modulating central carbon metabolism in *C. difficile*.

## RESULTS AND DISCUSSION

### Rationale for primary assay development

**Fig 2:**
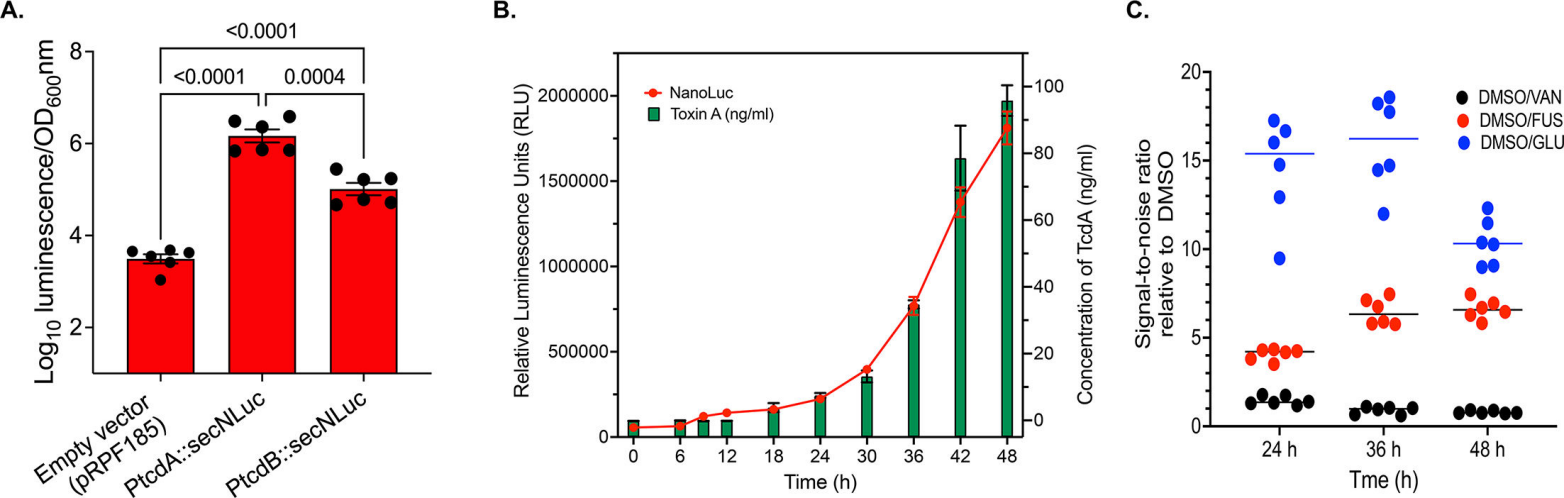
Validation of the reporter strain for the development of the primary screening assay. (**A**) Comparison of secNluc production in R20291 expressed from the promoters for *tcdA* or *tcdB*; statistical significance in GraphPad Prism 10.4.1 was by Welch’s one-way ANOVA with Dunnett’s T3 post-analysis test, and adjusted *P*-values are shown; biological replicates are shown. (**B**) Correlation between the production of secNluc and TcdA during the growth of the reporter strain R20291[PtcdA::secNluc]. The same supernatants were used to quantify secNluc activity (relative luminescence units) and TcdA amounts (ELISA). Data are from three biological replicates; Pearson correlation coefficient (*r* = 0.99, *P* < 0.0001, two-tailed) was determined in GraphPad Prism 10.4.1. (**C**) Effects of compounds on secNluc production in R20291[PtcdA::secNluc], based on signal/noise ratio at time points (24, 36, and 48 h). Logarithmic cultures (OD_600_ nm ~ 0.3) were exposed to DMSO, the negative control vancomycin (VAN; 0.4 µM), or the positive controls fusidic acid (FUS; 0.25 µM) and glucose (GLU; 1%, wt/vol). Luminescence was quantified from supernatants to determine the S/N ratio from three biological replicates, with each having a technical replicate (i.e., six data points).

We examined existing toxin quantification methods such as ELISA and cell rounding assays (13) but found them to be either semi-quantitative, costly, or too labor-intensive for large-scale, high-throughput primary screening of supernatants from cultures exposed to compounds. However, these methods were still valuable for orthogonal hit confirmation. Hence, we prioritized developing a primary screen using a reporter strain that produces a stable extracellular signal, correlating with TcdA and TcdB biosynthesis. Our initial attempts using beta-lactamase as the reporter were unsuccessful due to a low signal-to-noise ratio (*data not shown*). Therefore, we examined a reported codon-optimized secreted Nanoluc (secNluc) fused to the signal sequence of the zinc metalloprotease PPEP-1, resulting in its extracellular transport into culture media of strain *C. difficile* 630Δerm (24). We created two reporter strains in *C. difficile* R20291, an epidemic ribotype 027 (25), by cloning *secNluc* into vector pRPF185 under the *tcdA* or *tcdB* promoter. After 24-h growth in brain-heart infusion (BHI) broth in 96-well plates, culture supernatants from R20291[PtcdA::secNluc] exhibited luminescence signals 545.49 ± 279.3-fold higher than the empty vector control, while R20291[PtcdB::secNluc] showed only 37.9 ± 18.6-fold increase in luminescence (Fig. 2A). This is consistent with TcdB being the less produced of the two *C. difficile* toxins (20). R20291[*PtcdA::secNluc*] was, therefore, used to develop the primary screening assay. We next evaluated if the cellular production of secNluc correlates with TcdA production. As shown in Fig. 2B, the kinetics of secNluc production closely correlated with TcdA production measured by ELISA over a 48-h period, with a strong correlation (Pearson correlation coefficient, *r* = 0.99, *P* < 0.0001, two-tailed). Next, to examine the effects of inhibitors on signal intensity, we treated R20291[PtcdA::secNluc] cultures with positive controls glucose (1%, wt/vol) or sub-inhibitory concentrations of the protein synthesis inhibitor fusidic acid (0.25 µM), and a negative control, sub-inhibitory vancomycin (0.4 µM) (13, 26). Over 24 h, glucose and fusidic acid reduced secNluc activity (normalized to corresponding OD_600_nm) by 14.5 ± 2.9-fold and 4.06 ± 0.3-fold, respectively, relative to the DMSO control (Fig. 2C). Both agents continued to suppress secNluc production through 36 h of incubation, unlike vancomycin (Fig. 2C). However, by 48 h, the signal-to-noise ratio for vancomycin decreased, likely due to the natural death of vegetative *C. difficile* in late stationary phase cultures. Based on these results (Fig. 2B and C), we selected a 30-h incubation period for cultures in the 384-well screening assay to avoid the signal degenerating during longer incubation times caused by culture senescence.

### Screening of Prestwick compound library

Using the HTS assay guideline by the National Institutes of Health (27), we developed and validated a 384-well reporter assay using white µclear-bottom plates (Greiner Cat. No. 781098). Plates containing compounds in 5 µL of BHI were incubated anaerobically for 3 h before the addition of 45 µL of log-phase cells (OD_600_nm ~ 0.3 in BHI with 8 µg/mL thiamphenicol for plasmid maintenance). Plates were incubated for 30 h at 37°C. Plate uniformity in the 384-well format was evaluated using two experimental replicates, with DMSO (2%, wt/vol) and vancomycin (0.4 µM) as controls for high luminescence signals (negative controls) and fusidic acid (0.25 µM) and glucose (1%, wt/vol) as controls for low luminescence signals (positive controls). Results were analyzed using the Z-factor as a standard metric and the strictly standardized mean difference (SSMD) to examine assay variability due to biological heterogeneity that occurs in cell-based assays (28). The SSMD, which measures the signal-to-noise ratio by assessing the separation between positive and negative control signals relative to their variability, exceeded 4, indicating strong signal separation (Fig. S1). Although the Z′ scores were modest (0.26–0.37), which may be consistent with biological variability in *C. difficile* toxin production (29), the corresponding SSMD values (>4) indicated strong discrimination between the negative and positive control populations, supporting the use of the assay for HTS screening.

**Fig 3:**
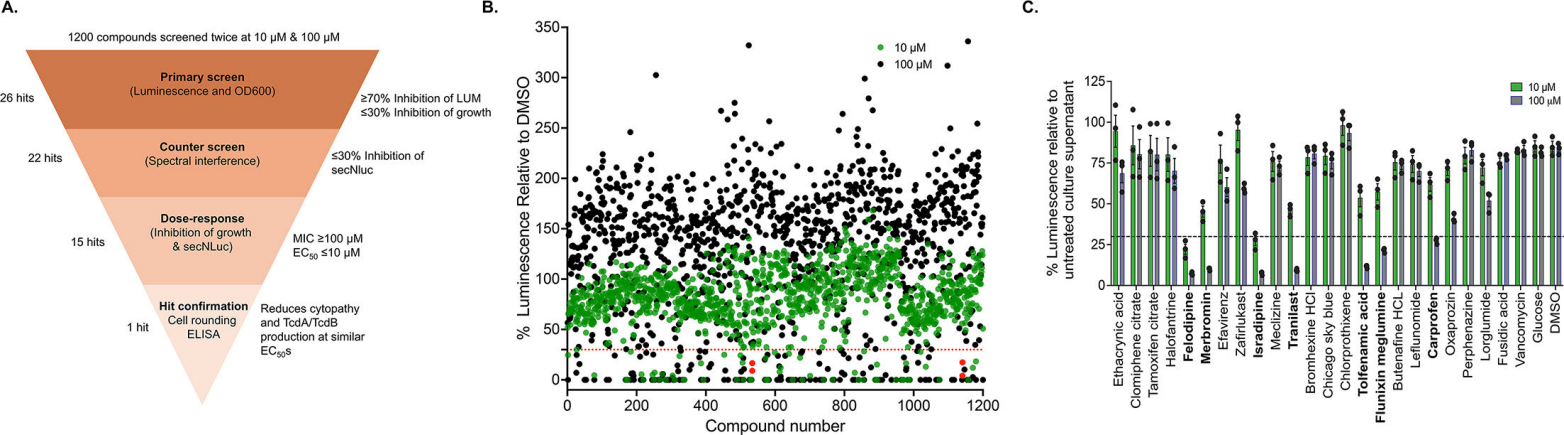
Screening of Prestwick Chemical Library. (**A**) Schematic of the workflow for screening 1,200 bioactives. Shown are the number of hits prioritized at each stage and the test criteria, leading to the selection of meclizine as the best hit. (**B**) Compounds were screened in 384-well plates against R20291[*PtcdA::secNluc*] in BHI broth. After 30 h of incubation, OD_600_nm and luminescence were measured. The scatter plot shows the percentage activity, normalized by culture ODs, with respect to DMSO. Each data point is the mean of two biological replicates. The dotted line indicates molecules reducing activity by >70% and included growth inhibitors. Meclizine and isradipine (red symbols at positions 534 and 1141, respectively) are hits common to the 10 and 100 µM screens. (**C**) Counter-screening for spectral interference was determined by detecting compounds inhibiting secNluc luminescence in culture supernatants from R20291[*PtcdA::secNluc*]; isradipine is a known inhibitor of NanoLuc. Data are from three biological replicates.

The Prestwick Chemical Library, comprising 1,200 bioactive compounds, was selected for preliminary screening because it includes major classes of both antibiotics and non-antibiotics. This diversity supports a critical assay strategy aimed at eliminating growth inhibitors and false positives, ultimately prioritizing candidate compounds that specifically reduce toxin synthesis (Fig. 3A). Each compound was tested at two logarithmic concentrations, 10 and 100 µM, to identify those that are potent at the lower concentration without inhibiting growth at the higher concentration. Compounds were selected based on two criteria: ≤30% inhibition of OD_600_nm (indicating minimal impact on bacterial growth) and ≥70% inhibition of luminescence activity (indicating strong suppression of toxin production). Based on these criteria, 26 compounds were identified as potential inhibitors of toxin biosynthesis across four screening rounds (Fig. 3B). Each screen yielded 14 hits (hit rate: 1.16%), including 12 unique compounds, while two compounds, isradipine and meclizine, were recovered in both screens (Fig. 3B; Table S1).

After excluding the antimicrobials dicloxacillin, metronidazole, ronidazole, and haloprogin, which did not significantly inhibit growth in the 10 µM screens, the remaining 22 non-antibiotic compounds were advanced to a series of hit confirmation assays (Fig. 3A and C). These included spectral interference testing, followed by orthogonal dose-response analyses to assess both growth inhibition (minimum inhibitory concentrations, MICs) and the potency of toxin synthesis inhibition (half-maximal effective concentrations, EC_50_ values) against R20291[PtcdA::secNluc]. Seven false positives were triaged as they inhibited secNluc luminescence by >70% when added to cell-free culture supernatants from untreated R20291[PtcdA::secNluc], which contained secNluc (Fig. 3C). This identified isradipine, a common hit in the 10 and 100 µM screens, as strongly quenching secNluc luminescence, which is consistent with it being a known inhibitor of the Nluc enzyme (27). For the remaining 15 hits, dose-responses revealed that hits from the 10 µM screen had lower EC_50_ values than those from the 100 µM screen (EC_50_s of 0.45–10.52 versus 10.45–79.82, respectively) (Table 1; Fig. S2a and b). In contrast, hits from the 100 µM screen did not show growth inhibition (MICs > 100 µM), whereas compounds from the 10 µM screen had MICs of 33.3 µM, with the exception of meclizine. Thus, meclizine emerged as a prioritized hit due to its strong inhibition of secNluc production (EC_50_ = 4.64 ± 0.41 µM) without affecting bacterial growth (MIC > 100 µM). After triaging for spectral interference, meclizine emerged as the top hit that demonstrated potency in reducing secNluc signals. As a result, meclizine was chosen for proof-of-principle studies to validate its activity as an inhibitor of toxin biosynthesis, thereby further demonstrating the effectiveness of the assay for identifying such molecules.

**TABLE 1:** Activities of hit compounds as determined by dose-responses*^a^*^,*b*^

Compound	Growth inhibition (MIC, μM)	Luminescence (EC_50_, μM)
Ethacrynic acid	33.3	0.45 ± 0.045
Clomiphene	33.3	7.15 ± 2.13
Tamoxifen	33.3	6.31 ± 1.19
Halofantrine	>100	6.40 ± 2.04
Efavirenz	100	10.52 ± 1.36
Zafirlukast	33.3–100	5.28 ± 0.78
Meclizine	>100	4.65 ± 0.41
Bromhexine	>100	54.99 ± 26.81
Butenafine	>100	10.45 ± 0.55
Lorglumide	>100	42.44 ± 29.12
Oxaprozin	>100	44.78 ± 21.33
Chlorprothixene	>100	79.82 ± 26.55
Leflunomide	>100	67.46 ± 80.09
Perphenazine	>100	79.20 ± 37.71
Chicago Sky Blue 6B	>100	>100

^*a*^ R20291[PtcdA::secNluc] was used to measure growth inhibition and luminescence from the same wells.

^*b*^ Shown are inhibition of growth (MIC) and secNluc production (luminescence).

### Confirmation of meclizine as an inhibitor of toxin biosynthesis

**Fig 4:**
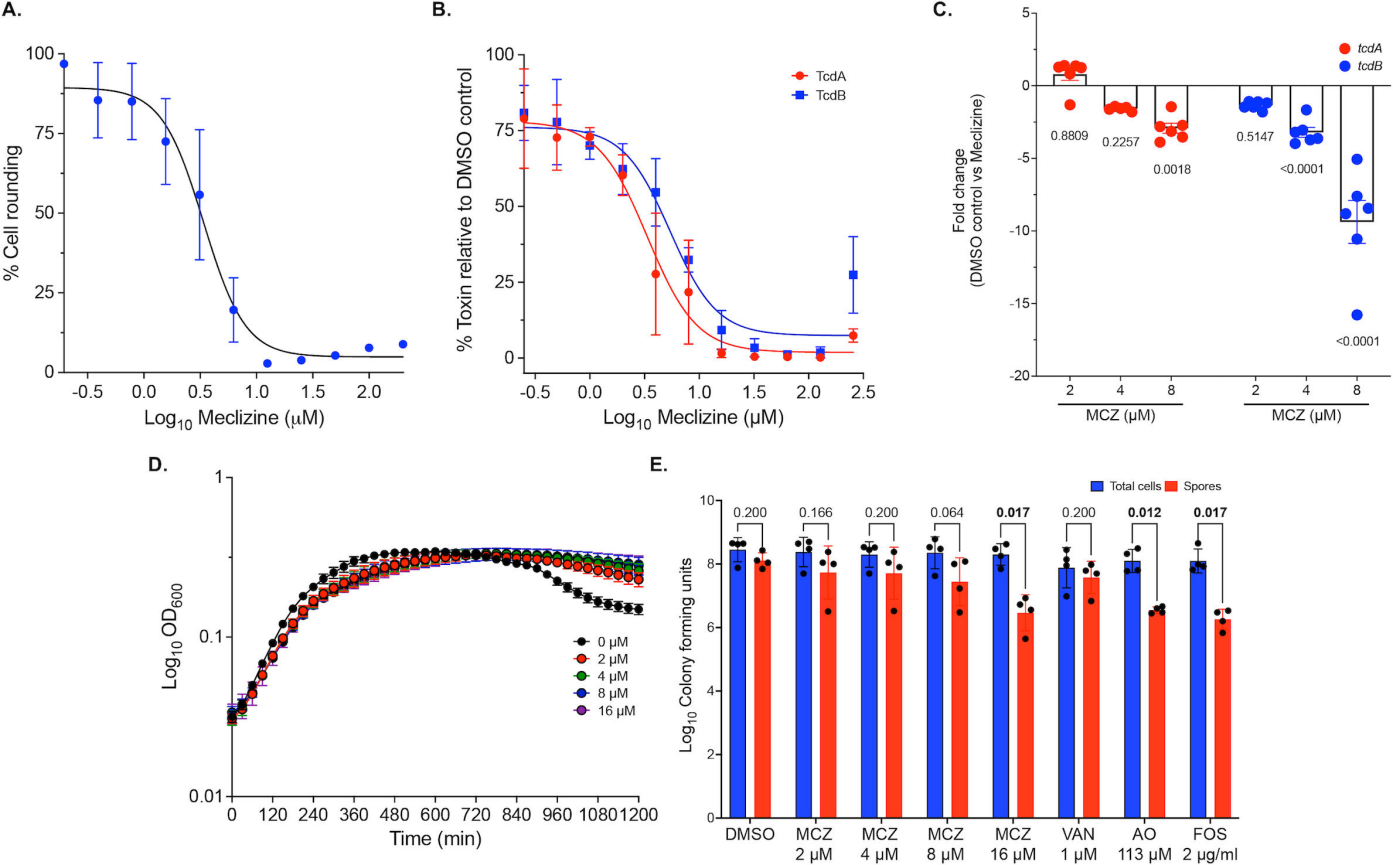
Characterization of the antivirulence activity of meclizine (MCZ) against *C. difficile*. (**A and B**) Quantification of meclizine’s inhibition of toxin production in strain R20291. Logarithmic cultures (OD_600_nm ~ 0.3) were treated with twofold concentrations of meclizine, and toxin levels were measured after 24 h by cytopathic cell rounding assay (**A**) and ELISA (**B**); data are representative for three biological replicates. (**C**) mRNA levels of *tcdA* and *tcdB* were analyzed by RT-qPCR after exposing logarithmic cultures (OD_600_nm ~ 0.3) to varying concentrations of meclizine for 9 h; fold changes were determined relative to mRNA from the DMSO control. Statistical analysis was assessed from the ΔCt values of the control and meclizine-treated cultures by one-way ANOVA with Tukey’s post-analysis test and shown as adjusted *P* values from GraphPad Prism 10.4.1. (**D**) Growth of R20291 (OD_600_nm ~ 0.2) following treatment with DMSO or meclizine (0.5, 1, 2, or 4 times the EC_50_ value) from six biological replicates. (**E**) Effect of meclizine on sporulation over 5 days. Cultures (OD_600_nm ~ 0.3; from three biological replicates). Heat-resistant spores were enumerated from the total cell population. Compounds were meclizine (0.5, 1, 2, or 4 times the EC_50_ value), vancomycin (VAN) at 0.5 µM, acridine orange (AO) at 116 µM, or fosfomycin (FOS) at 1.45 µM. Data from four biological replicates are shown as mean ± standard mean of error; statistical significance was assessed by multiple paired *t*-tests with alpha set to 0.05 and multiple comparisons correction using the Holm-Šídák method and shown as adjusted *P* values in GraphPad Prism 10.4.1.

To validate meclizine as an inhibitor of toxin biosynthesis, wild-type *C. difficile* R20291 was exposed to the compound for 24 h, and supernatants were analyzed for cytotoxicity using cell rounding assays and TcdA and TcdB levels via ELISA. Meclizine had an EC_50_ of 3.38 µM in the cell rounding assay (Fig. 4A) and effectively inhibited TcdA and TcdB production with EC_50_ values of 3.35 and 5.42 µM, respectively (Fig. 4B). Based on these results, 4 µM was selected as an approximate EC_50_ for use in subsequent experiments. Transcriptional analysis by RT-qPCR on *C. difficile* R20291 exposed to meclizine for 9 h revealed dose-dependent inhibition of toxin gene expression (Fig. 4C). Specifically, *tcdB* mRNA levels were reduced by 3.2 ± 0.83-fold and 9.37 ± 3.60-fold at 4 and 8 µM, respectively. While *tcdA* mRNA levels were not significantly affected at 4 µM, they were reduced by 2.92 ± 0.84-fold at 8 µM. Against other major CDI-associated ribotypes (001, 020, 002, 078, and 106), meclizine exhibited EC_50_ values ranging from 4.80 to 16.70 µM, as determined by the cell rounding assay (Fig. S3).

### Effect of meclizine on bacterial growth

Growth curve analysis of *C. difficile* R20291, based on OD_600_nm measurements, showed that meclizine did not inhibit bacterial growth at concentrations up to 16 µM, four times the adopted EC_50_ for toxin biosynthesis, indicating that its inhibitory effect on toxin production is not due to antimicrobial activity (Fig. 4D). Notably, during the stationary phase (810 min), meclizine-treated cultures maintained higher cell densities compared to DMSO-treated controls, suggesting a potential metabolic difference (Fig. 4D). Furthermore, meclizine exhibited no antimicrobial activity against a panel of gut microbial species, with MICs ≥ 128 µM (Table 2), implying minimal impact on the gut microbiota species.

**TABLE 2:** Comparison of antimicrobial activities of meclizine and vancomycin against gut flora

Species/strain	Activity (μg/mL)	
	Meclizine	Vancomycin
*Bacteroides eggerthii* HM210	>128	<2
*Bacteroides fragilis* HM20	>128	8
*Bacteroides ovatus* HM222	>128	16
*Bacteroides* sp. HM18	>128	32
*Bacteroides* sp. HM19	>128	128
*Bacteroides* sp. HM23	>128	64
*Bacteroides* sp. HM28	>128	64
*Lactobacillus crispatus* HM421	>128	16
*Fusobacterium nucleatum* HM260	128	>128
*Lactobacillus johnsonii* HM643	>128	4
*Porphyromonas uenonis* HM130	128	4

### Effect of meclizine on sporulation

Besides toxin production, sporulation is an important factor influencing the pathogenesis of CDI, as spores are responsible for disease transmission, and their survival in the gut during antibiotic treatment plays a role in endogenous recurrence of the disease during persisting dysbiosis (30). Furthermore, since there is regulatory interplay between toxin production and sporulation pathways in *C. difficile*, we investigated whether meclizine affects sporulation (17, 18). R20291 cultures were exposed to doubling concentrations of meclizine (2–16 μM), spanning from half to four times the EC_50_ for toxin biosynthesis. After 5 days, cultures treated with 16 µM meclizine showed a 2-log reduction in spore burden without affecting total viable counts (Fig. 4E).

### Meclizine alters carbohydrate metabolism, increasing cellular glucose and pyruvate levels

**Fig 5:**
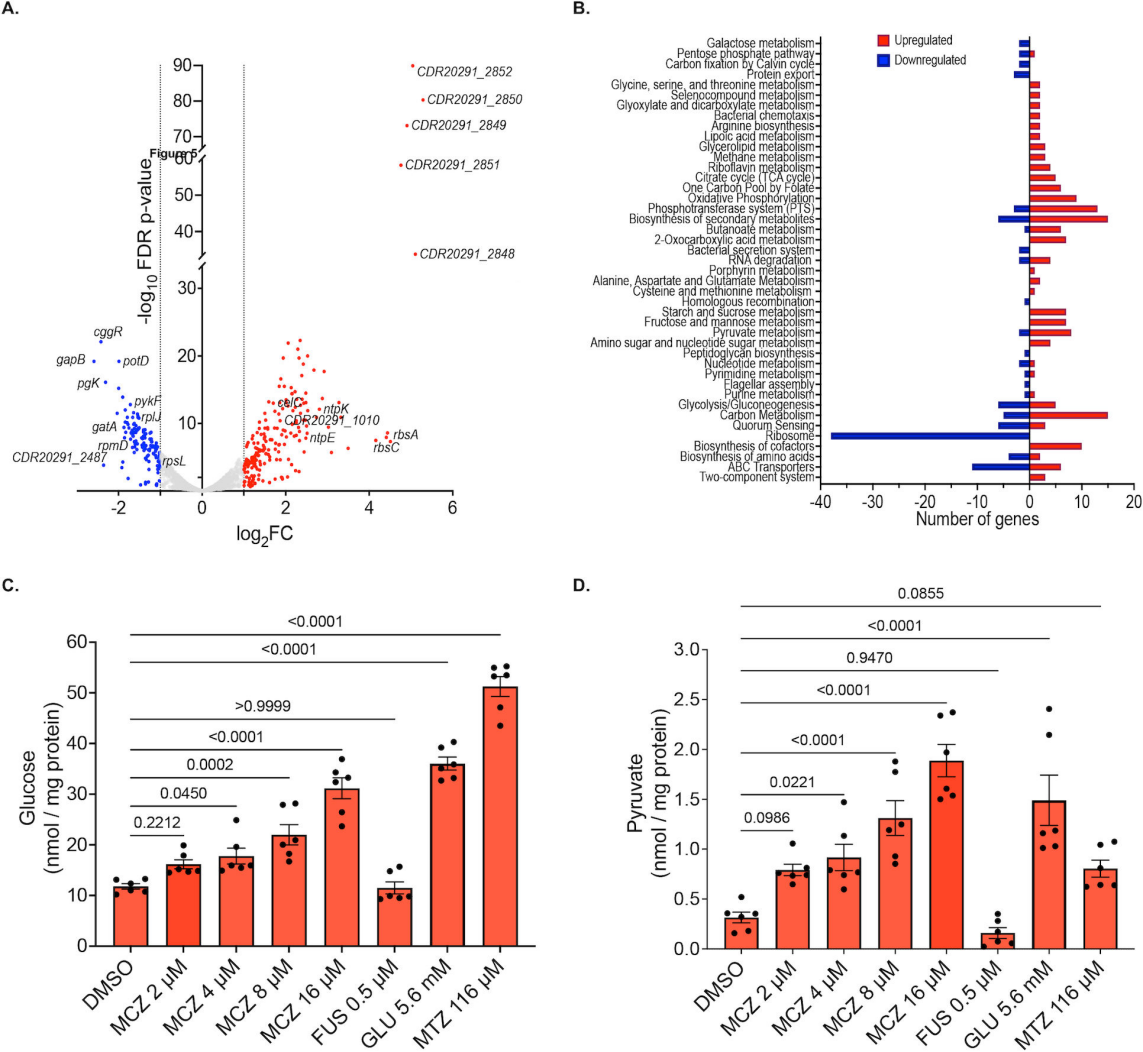
Physiological response of *C. difficile* to meclizine (MCZ). (**A**) Transcriptional response of R20291 to meclizine, following exposure of mid-logarithmic cells (OD_600_nm ~ 0.5) to meclizine (8 µM) for 1 h. The volcano plot shows statistical significance of differentially expressed genes that were upregulated (red symbols) or downregulated (blue symbols) compared to DMSO. (**B**) KEGG pathway analysis of metabolic pathways altered by meclizine. (**C and D**) Changes in the cytosolic content of glucose (**C**) and pyruvate (**D**) in logarithmic cultures (OD_600_nm ~ 0.3) exposed to DMSO, meclizine (0.5, 1, 2, or 4 times the EC_50_ value), metronidazole (116 µM), or glucose (5.6 mM). Glucose and pyruvate were analyzed from whole cell lysates (*n* = 3 biological replicates). Statistical significance was assessed by one-way ANOVA with Dunnett’s post-analysis test and shown as adjusted *P* values in GraphPad Prism 10.4.1.

To further investigate meclizine’s mechanism of action, transcriptomic profiling was performed on R20291 cultures (OD_600_nm = 0.3) exposed to 8 µM meclizine for 1 h. RNA-seq analysis identified 292 differentially expressed genes (DEGs), including 172 upregulated and 120 downregulated transcripts (Fig. 5A; Table S2). DEGs were defined by a log2 fold change ≥ 1.0 (equivalent to a ≥2-fold change) and a false discovery rate (FDR) ≤ 0.01. Expression patterns of selected genes validated by RT-qPCR showed strong concordance with RNA-seq data (Pearson’s correlation coefficient *r* = 0.78, *P* = 0.0003; Fig. S4). Functional clustering of DEGs using KEGG Mapper and STRING-DB revealed that meclizine significantly impacted multiple toxin-associated pathways (Fig. 5B; Table 3).

**TABLE 3:** Selection of genes that were differentially expressed^*a*^ in *C. difficile* R20291 following exposure to meclizine^*b*^

Functional group/gene ID	Encoded proteins	Log_2_ fold change
**Upregulated genes**		
Sugar transport and metabolism		
*CDR20291_2848–CDR20291_2852*	Putative glycosyl hydrolase; fructose-like phosphotransferase system (PTS) system IIabc component; putative transcriptional antiterminator	4.76–5.29
*CDR20291_2863–CDR20291_2865*	Putative phosphosugar isomerase, putative bifunctional protein: repressor/cystathionine beta-lyase, PTS system, maltose and glucose-specific IIbc component	1.59–2.50
*CDR20291_2971*	PTS system IIabc component, glucose/maltose/N-acetylglucosamine-specific; PgmB: beta-phosphoglucomutase; putative glycosyl hydrolase	1.01–2.39
*CDR20291_2554, CDR20291_2555*	Crr: PTS system, glucose-specific IIa component; PtsG: PTS system, glucose-specific IIbc component	0.84–1.00
*CDR20291_2220, CDR20291_2221*	MtlD: mannitol-1-phosphate 5-dehydrogenase; MtlF: predicted PEP-driven transporter of D-mannitol	2.32–2.37
*CDR20291_2926 – CDR20291_2928*	Phosphosugar-binding transcriptional regulator; putative cellobiose-phosphate degrading protein; PTS system, IIabc component	1.01–2.01
*CDR20291_2914, CDR20291_2915*	TagT: PTS system, IIabc component; TagK: putative tagatose (1)-phosphate kinase	1.85–1.87
*CDR20291_2776 – CDR20291_2780*	CelC: PTS system, lichenan-specific IIa component; conserved hypothetical protein; CelF: 6-phospho-beta-glucosidase; CelB PTS system, lichenan-specific IIc component; LicB: PTS system, lichenan-specific IIb component	1.24–1.74
Glycine fermentation		
*CDR20291_2237–CDR20291_2238*	GrdD, GrdC: glycine reductase subunits D, C	2.31–2.74
*CDR20291_2240–CDR20291_2244*	GrdA, GrdE: glycine reductase subunits A, E; TrxA2: thioredoxin; TrxB3: thioredoxin reductase; GrdX: putative glycine reductase complex component	2.23–2.92
Bifurcation/confurcation (pyruvate formation)		
*CDR20291_1007–CDR20291_1010*	LarA: lactate racemase; EtfB3, EtfA3: electron transfer flavoprotein beta and alpha subunits; FAD/FMN-containing lactate dehydrogenase	1.87–2.47
V-type sodium ATP synthase		
*CDR20291_2787–CDR20291_2794*	V-type sodium ATP synthase subunits D, B, A, G, C, E, K, and I	1.94–2.43
**Downregulated genes**		
Glycolysis		
*CDR20291_3216*	PykF: pyruvate kinase	−1.72
*CDR20291_3030*	GapB: glyceraldehyde-3-phosphate dehydrogenase 2	−2.59
*CDR20291_3031*	CggR: central glycolytic genes regulator	−2.42
*CDR20291_3029*	Pgk: phosphoglycerate kinase	−2.31
*CDR20291_2487*	CstA: putative carbon starvation (pyruvate transporter)	−2.36
Ribosomal protein synthesis		
*CDR20291_0062–CDR20291_0096*	Gene cluster encoding proteins involved in protein synthesis at the ribosomal stage; rpsJ, rplC, rplD, rplW, RplB, RpsS, RplV, RpsC, RplP, RpmC, RpsQ, RplN, RplX, RplE, RpsZ, RpsH, RplF, RplR, RpsE, RpmD, RplO, PrlA, Adk, Map1, RpmJ, RpsM, RpsK, RpsD, RpoA	−1.05 to −1.90

^*a*^ Values are shown as the range for the log2 fold change. Selected genes indicate key pathways modulated by meclizine from among those observed in the RNA-seq (Table S2). *CDR20291_2852* was the most upregulated gene, while *gapB* was the most downregulated.

^*b*^ Functional classifications are shown.

### Upregulated genes

Significantly upregulated genes included those involved in energy generation and redox, such as ethanolamine utilization, glycine, and leucine fermentation via the Stickland pathway, and the V-type sodium ATP synthase (Table 3). Notably, there was upregulation of the operon-containing genes involved in confurcation (29), a metabolic pathway converting lactate to pyruvate (i.e., expression changes for genes in the operon ranged from log2 = 1.87–2.47, corresponding to 3.66- to 5.54-fold). This operon encodes lactate racemase (*larA; CDR20291_1007*), electron transfer flavoproteins (*etfA3B3; CDR20291_1008* to *CDR20291_1009*), and an FAD/FMN-containing lactate dehydrogenase (*CDR20291_1010*) (31). *C. difficile* possesses a large repertoire of phosphotransferase system (PTS) sugar transporters (18), many of which were among the most highly upregulated genes in the RNA-seq data. For example, the gene cluster encoding the fructose-like specific transporter enzyme IIABC components (*CDR20291_2849* to *CDR20291_2851*) and the PTS transcriptional anti-terminator (*CDR20291_2852*) were highly upregulated, whereas a putative glycosyl hydrolase, *CDR20291_2848*, was upregulated by 34.30-fold. Other significantly upregulated PTS system transporters included *CDR20291*_2971, *pgmB,* and *CDR20291*_2973, encoding a PTS system IIABC component, a beta-phosphoglucomutase, and a putative glycosyl hydrolase, respectively. Additionally, *CDR20291_2927* and *CDR20291_2928*, encoding a putative cellobiose-phosphate-degrading protein and PTS system IIABC component related to the glucose transporter PtsG, respectively, were also upregulated. These findings suggest that meclizine altered the uptake and metabolism of carbohydrates.

### Downregulated genes

Interestingly, while meclizine did not attenuate logarithmic *C. difficile* growth, it downregulated at least 45 genes involved in protein biosynthesis, such as *rpsN* (encoding 30S ribosomal protein S14), elongation factor TU (*CDR20291_0065*), and translation initiation factors IF-1 (*CDR20291_0091*) and IF-3 (*CDR20291_0611*). The most downregulated genes are involved in glycolysis and pyruvate metabolism, including *cggR* (*CDR20291_3031*, central glycolytic genes regulator); *gapB* (*CDR20291_3030*, glyceraldehyde-3-phosphate dehydrogenase 2); *CDR20291_2487* (putative carbon starvation protein CstA); *pgK* (*CDR20291_3029*, phosphoglycerate kinase); *pfkA* (*CDR20291_3217*, 6-phosphofructokinase); *pykF* (*CDR20291_3216*, pyruvate kinase)*;* and *adhE* (*CDR20291_0339*, aldehyde-alcohol dehydrogenase [pyruvate formate lyase]). These findings may indicate that *C. difficile* altered ribosome production and its translational machinery to cope with less favorable metabolic conditions induced by meclizine and that meclizine reduced the expression of genes involved in central carbon metabolism.

### Cellular build-up of glucose and pyruvate molecules

Given that the RNA-seq data suggested meclizine disrupted central carbon metabolism, potentially leading to the accumulation of glucose and pyruvate, both known to suppress TcdA and TcdB production, we quantified these metabolites intracellularly in R20291 grown in BHI and exposed to meclizine (2–16 μM) for 3 h. A dose-dependent increase in intracellular glucose and pyruvate was observed: glucose levels rose by 1.40- to 2.67-fold (40%–156%), while pyruvate levels increased by 3.30- to 7.01-fold (230%–600%) (Fig. 5C and D). The intracellular glucose likely originated from the BHI medium, which contains 0.2% (wt/vol) glucose, whereas pyruvate was likely synthesized endogenously. As expected, supplementation with 1% (wt/vol) glucose (positive control) elevated intracellular glucose and pyruvate levels by 3.07-fold (207%) and 6.44-fold (544%), respectively. Metronidazole and fusidic acid were also included as positive and negative controls. Metronidazole significantly elevated glucose and pyruvate levels (4.39- and 2.87-fold, respectively), consistent with its mode of action involving reduction by oxidoreductases such as pyruvate:ferredoxin oxidoreductase, generating free radicals that disrupt energy metabolism (26). In contrast, fusidic acid, a protein synthesis inhibitor, had no such effect.

### Concluding remarks

Drug discovery efforts targeting the pathogenesis of *C. difficile* have primarily focused on inhibiting TcdA and TcdB using biologics (e.g., bezlotoxumab) or small molecules. To broaden antivirulence therapeutic strategies against *C. difficile*, we developed a new HTS platform to identify inhibitors of toxin biosynthesis. Although limited in size (~1,200 compounds), the Prestwick library provided an ideal proof-of-concept data set encompassing chemically and mechanistically diverse FDA-approved molecules, allowing evaluation of the assay’s robustness before scaling to larger, structurally diverse collections. It enabled implementation of a critical path for compound progression, involving triaging of most known antibacterials, counter-selection for spectral interference, and hit prioritization based on potency and lack of growth inhibition in dose-response assays. While adoption of the SSMD statistical analysis indicated strong assay robustness, further optimization of the primary screen is needed to enhance secNluc signal intensity, to improve the dynamic range of the controls, and achieve *Z*′ > 0.5 for larger-scale screens of thousands of compounds. Improvements may include optimizing culture conditions to increase *tcdA* promoter activity and evaluating alternative plate readers to enhance signal detection. Meclizine was selected for proof-of-principle studies to further validate the utility of the screening platform. Findings showed that it inhibited TcdA and TcdB biosynthesis and sporulation. However, we must emphasize that additional hit molecules in Table 2 may still be candidates for follow-up studies, such as drug repurposing or molecular probes to discover antivirulence drug targets in *C. difficile*. Noteworthy, while the assay was developed to identify inhibitors of toxin biosynthesis, this assay format may also be applicable to finding compounds with anti-sporulation activity since these two pathways have regulatory overlaps (17, 18). For example, we reported that both toxin production and sporulation were inhibited by enoxolone (13), a phytochemical with multi-targeted action against ATP synthase, phosphate, and the purine salvage in *C. difficile*. Toxin biosynthesis and sporogenesis are co-repressed by the catabolite repressor protein CcpA through metabolizable sugars, such as glucose (18–20), while pyruvate has been shown to repress toxin production (22). Hence, our finding that meclizine, discovered as a toxin synthesis inhibitor, also inhibits sporulation is consistent with these pathways being linked. While it is plausible that meclizine’s disruption of central carbon metabolism is a potential underlying mechanistic reason for inhibiting both toxin biosynthesis and sporulation, the precise molecular targets of meclizine need to be defined. Therefore, future studies are aimed at dissecting the precise mechanisms and targets by which meclizine perturbs *C. difficile* metabolism to inhibit both toxin biosynthesis and sporulation. Such mechanistic insights will guide the rational design of meclizine analogs with greater drug development potential and determine the extent to which their cellular actions contribute to efficacy in animal models of CDI.

## MATERIALS AND METHODS

### Strains and growth conditions

*C. difficile* strains were routinely cultured in BHI medium with 0.1% (wt/vol) taurocholate or 15 µg/mL thiamphenicol for plasmid maintenance when required. Gut microbial species from Biodefense and Emerging Infectious Research Resource Respiratory (Manassas, VA, USA) at the American Type Culture Collection (Manassas, VA, USA) were routinely grown in brucella agar or broth supplemented with 5% (vol/vol) defibrinated sheep blood (Hardy Diagnostics), 5 mg/L hemin, and 10 mg/L vitamin K1. All strains were cultured anaerobically in a Don Whitley A35 anaerobic chamber at 37°C. *E. coli* was grown at 37°C aerobically in Luria Bertani agar or broth medium supplemented with 35 µg/mL of chloramphenicol or 50 µg/mL of kanamycin when required.

### Genetic construction of reporter strains

R20291[PtcdA::secNluc] and R20291[PtcdB::secNluc] were constructed using codon-optimized nano-luciferase gene fused to the PPEP-1 signal sequence at the N-terminus (GenBank accession no. AF179295.1). The gene was synthesized by GenScript Biotech (Piscataway, NJ, USA), digested with SacI-BamHI, and cloned into similarly digested pRPF185 (32), generating pRPF185-secNluc. The promoters for *tcdA* and *tcdB* of *C. difficile* R20291 were amplified, with flanking KpnI-SacI restriction sites, digested, and cloned into similarly digested pRPF185-secNluc to yield pRPF185-secNluc-P*_tcdA_* and pRPF185-secNluc-P*_tcdB_*, respectively. The plasmids were conjugated into R20291 from *E. coli*, producing the two reporter strains.

### Measurement of luciferase activity

Single colonies of R20291 were inoculated into 3 mL of BHI broth. The resulting overnight culture was diluted 100-fold with fresh pre-reduced BHI and grown until OD_600_ ~ 0.2–0.3 before compounds were added to the desired concentration. At time points, supernatants were recovered from 1 mL samples that were centrifuged at 21,000 × *g* for 1 min. Luciferase activity was measured by mixing culture supernatant and NanoGlo luciferase substrate (Promega Cat. No. N1110) and measuring luminescence using a Cytation5 plate reader from Biotek; luminescence readings were normalized according to culture OD_600_nm.

### HTS screening

The Prestwick library of 1,200 bioactive molecules was screened in a 384-well format using white µclear bottom plates (Greiner Cat. No. 781098). Stock plates containing 10 mM of compounds in 100% DMSO were stored at −20°C. Stock plates were thawed, and compounds were dispensed into the 384-well plates using the Echo acoustic liquid handler (Labcyte). Each well also contained 5 µL of BHI. Plates were incubated in a Don Whitley A35 anaerobic chamber. Meanwhile, overnight cultures of R20291[PtcdA::secNluc] were diluted 100-fold in fresh BHI broth and grown anaerobically to OD_600_ ~ 0.3, before 45 µL was dispensed into the above 384-well plates using a Multidrop Combi Reagent Dispenser from Thermo Fisher Scientific. Plates were incubated for 30 h, and optical densities (OD_600_nm) were measured using a Cytation5 microplate reader. Next, 5 µL of NanoGlo luciferase substrate was added to the plates, and luminescence was measured in the same Cytation5 microplate reader. Luminescence readings were normalized to the corresponding OD_600_nm values.

### Cell rounding assay

Assessment of the cytopathic effect of supernatants, as a measure of toxin production, was performed using Vero epithelial cells (ATCC), which are more sensitive to toxin B (13, 33). Briefly, 50 µL of Vero cells was seeded into 384-well plates to a final density of 10^4^ cells/mL and incubated overnight at 37°C in a CO_2_ incubator. Using an Echo acoustic liquid handler (Labcyte), culture supernatants (100 nL) were added to the Vero cells and incubated for 3 h, before being fixed with 4% (vol/vol) paraformaldehyde and stained with 4′,6-diamidino-2-phenylindole (DAPI) and Alexa Fluor 488 phalloidin. Images were acquired with the IN Cell Analyzer 6000 (GE Healthcare) for DAPI (excitation 405 nm; emission 455 nm) and phalloidin (excitation 495 nm; emission 515 nm). Images were quantified using a custom analysis built using tools from the “Imaging” and “Analysis and Statistics” libraries in Pipeline Pilot version 9.2 (Biovia). Here, images were background corrected, segmented using morphological operators, and a panel of morphometric features was quantified. A random forest analysis was then used to classify cells into control-like or toxin-treated-like (small, rounded) conditions, and the proportion of toxin-treated-like cells per well was determined as described previously (13).

### ELISA detection of toxins

The amount of TcdA and TcdB in culture supernatants was analyzed using the *C. difficile* toxin A or B ELISA Kit (tgcBIOMICS) according to the manufacturer’s instructions.

### Measurement of luminescence quenching

Overnight cultures of R20291[PtcdA::secNluc] were diluted 100-fold in fresh BHI medium and allowed to grow for 48 h. Cultures were harvested by centrifugation at 4,000 × *g* for 5 min, and the resulting supernatants were passed through 0.22 µm filters. In 96-well plates, supernatants were incubated with test compounds at 10 and 100 µM for 1 h. Luminescence activity was measured by mixing 190 µL of the test sample with 10 µL of NanoGlo luciferase substrate and recorded in the Cytation5 plate reader (Biotek).

### Growth kinetics and inhibition

Minimal inhibitory concentrations were determined as previously described (13) by microbroth dilution in BHI broth in 96-well microtiter plates. Following anaerobic incubation for 24 h, the MIC was defined as the lowest concentration of compound inhibiting visible growth. To assess the effect of compounds on bacterial growth kinetics, overnight cultures were diluted 100-fold in BHI broth and grown to OD_600_nm ~ 0.2. Cultures were then added to 96-well plates containing twofold serial dilutions of test compounds. Optical densities (OD_600_nm) were continuously recorded over a 20-h period in an Infinite M Plex microplate reader (Tecan) in an anaerobic chamber (Coy Laboratory Products).

### Sporulation

The effect of compounds on sporulation was performed essentially as described (13). Briefly, single colonies of R20291 grown on BHI agar with 0.1% (wt/vol) taurocholate were inoculated in fresh BHI broth and grown to OD_600_nm ~ 0.3. After adding compounds, cultures were incubated for 5 days, followed by enumerating total viable cells and heat-resistant spores by plating serial dilutions onto BHI agar with 0.1% (wt/vol) taurocholate, using a WASP automated spiral plater (Don Whitley Scientific).

### Transcriptome analysis

Cultures were grown to OD_600_nm ~ 0.3, followed by treatment with DMSO or meclizine (8 µM). After 1 h, cultures were treated with one volume of RNAprotect (Qiagen) followed by centrifugation (4,000 × *g* for 10 min). Total RNA was extracted using the RNeasy Mini Kit (Qiagen) according to the manufacturer’s instructions. Assessment of RNA quality, library preparation, and sequencing were performed by SeqCenter (Pittsburgh, PA, USA). Bioinformatic analysis was done by uploading raw FASTQ files onto the Galaxy platform (https://usegalaxy.org). Quality control and trimming were done with FastQC and Trim Galore, respectively. Using BWA-MEM, processed reads were aligned to the R20291 reference genome (accession number FN545816). Counts per read were generated using HTSeq-count, and the count matrix was generated with the Column Join tool. A differential gene expression file was generated by analyzing the count matrix file in Degust (https://degust.erc.monash.edu/) using edgeR (cutoffs, fold change of 2.0, and FDR of 0.01).

### Quantitative reverse-transcription analysis

RNA was extracted as above, and cDNA was prepared from 1 µg of total RNA using qScript cDNA Supermix (Quanta Biosciences). Quantitative PCR was done with Sso Advanced Universal SYBR Green Supermix (BioRad) in ViiA7 RT-PCR System (Applied Biosystems). Fold changes in the transcript levels were calculated using the threshold cycle (*C_T_*) values by the 2^ΔΔC^_T_ method, and transcripts were normalized to 16S rRNA.

### Measurement of intracellular glucose and pyruvate

Fresh BHI was inoculated with an overnight culture at 1% (vol/vol) and incubated anaerobically to OD_600_nm ~ 0.3. Cultures were aliquoted into fresh tubes with compounds at the required concentrations, and cultures were grown for an additional 3 h. After harvesting cultures at 4,000 × *g* for 10 min, cell pellets were washed twice with ice-cold Milli-Q water and lysed by bead beating in a Qiagen TissueLyser LT, with precooling of the sample holder prior to use. Pyruvate was quantified in lysates using the Sigma-Aldrich Pyruvate Assay Kit according to the manufacturer’s instructions. Glucose was quantified in lysates using Promega’s Glucose-Glo Assay Kit according to the manufacturer’s instructions. Data was normalized according to the protein content in samples.

## Data Availability

Data described in this study include assay development, screening results, dose-response curves, MICs, RNA-seq data (shown in Tables 1 to 3 and Tables S1 and S2; and Fig. 2 to 4; Fig. S1 to S4; and the main text). Figure 5 describes cellular mode of action data confirming findings from the RNA-seq; Fig. S4 shows validation of the RNA-seq by RT-qPCR. The RNA-seq data are deposited in NCBI under BioProject number PRJNA1279663.

## SUPPLEMENTAL MATERIAL

Supplemental figures aac.00960-25-s0001.pdf Figures S1 to S4.

Table S1 aac.00960-25-s0002.pdf Screening results for Prestwick chemical library.

Table S2 aac.00960-25-s0003.xlsx Significantly transcribed genes in *C. difficile* R20291 in response to meclizine compared to untreated controls.

ASM does not own the copyrights to Supplemental Material that may be linked to, or accessed through, an article. The authors have granted ASM a non-exclusive, world-wide license to publish the Supplemental Material files. Please contact the corresponding author directly for reuse.

## References

[1] Lessa FC, Mu Y, Bamberg WM, Beldavs ZG, Dumyati GK, Dunn JR, Farley MM, Holzbauer SM, Meek JI, Phipps EC, Wilson LE, Winston LG, Cohen JA, Limbago BM, Fridkin SK, Gerding DN, McDonald LC. 2015. Burden of Clostridium difficile infection in the United States. N Engl J Med 372:825–834. doi:10.1056/NEJMoa1408913 25714160 PMC10966662

[2] Ghantoji SS, Sail K, Lairson DR, DuPont HL, Garey KW. 2010. Economic healthcare costs of Clostridium difficile infection: a systematic review. J Hosp Infect 74:309–318. doi:10.1016/j.jhin.2009.10.016 20153547

[3] Lewis BB, Buffie CG, Carter RA, Leiner I, Toussaint NC, Miller LC, Gobourne A, Ling L, Pamer EG. 2015. Loss of microbiota-mediated colonization resistance to Clostridium difficile infection with oral vancomycin compared with metronidazole. J Infect Dis 212:1656–1665. doi:10.1093/infdis/jiv256 25920320 PMC4621244

[4] Zhang Y, Limaye PB, Renaud HJ, Klaassen CD. 2014. Effect of various antibiotics on modulation of intestinal microbiota and bile acid profile in mice. Toxicol Appl Pharmacol 277:138–145. doi:10.1016/j.taap.2014.03.009 24657338 PMC5533088

[5] Guery B, Menichetti F, Anttila V-J, Adomakoh N, Aguado JM, Bisnauthsing K, Georgopali A, Goldenberg SD, Karas A, Kazeem G, Longshaw C, Palacios-Fabrega JA, Cornely OA, Vehreschild M, EXTEND Clinical Study Group. 2018. Extended-pulsed fidaxomicin versus vancomycin for Clostridium difficile infection in patients 60 years and older (EXTEND): a randomised, controlled, open-label, phase 3b/4 trial. Lancet Infect Dis 18:296–307. doi:10.1016/S1473-3099(17)30751-X 29273269

[6] Louie TJ, Miller MA, Mullane KM, Weiss K, Lentnek A, Golan Y, Gorbach S, Sears P, Shue Y-K, OPT-80-003 Clinical Study Group. 2011. Fidaxomicin versus vancomycin for Clostridium difficile infection. N Engl J Med 364:422–431. doi:10.1056/NEJMoa0910812 21288078

[7] Monaghan TM, Seekatz AM, Mullish BH, Moore-Gillon CCER, Dawson LF, Ahmed A, Kao D, Chan WC. 2021. Clostridioides difficile: innovations in target discovery and potential for therapeutic success. Expert Opin Ther Targets 25:949–963. doi:10.1080/14728222.2021.2008907 34793686 PMC12107399

[8] Gonzales-Luna AJ, Carlson TJ, Garey KW. 2023. Emerging options for the prevention and management of Clostridioides difficile infection. Drugs (Abingdon Engl) 83:105–116. doi:10.1007/s40265-022-01832-x PMC9841950 36645620

[9] Chandrasekaran R, Lacy DB. 2017. The role of toxins in Clostridium difficile infection. FEMS Microbiol Rev 41:723–750. doi:10.1093/femsre/fux048 29048477 PMC5812492

[10] Gerding DN, Johnson S. 2010. Management of Clostridium difficile infection: thinking inside and outside the box. Clin Infect Dis 51:1306–1313. doi:10.1086/657116 20979491

[11] Gerding DN, Kelly CP, Rahav G, Lee C, Dubberke ER, Kumar PN, Yacyshyn B, Kao D, Eves K, Ellison MC, Hanson ME, Guris D, Dorr MB. 2018. Bezlotoxumab for prevention of recurrent Clostridium difficile infection in patients at increased risk for recurrence. Clin Infect Dis 67:649–656. doi:10.1093/cid/ciy171 29538686 PMC6093994

[12] Paparella AS, Aboulache BL, Harijan RK, Potts KS, Tyler PC, Schramm VL. 2021. Inhibition of Clostridium difficile TcdA and TcdB toxins with transition state analogues. Nat Commun 12:6285. doi:10.1038/s41467-021-26580-6 34725358 PMC8560925

[13] Marreddy RKR, Phelps GA, Churion K, Picker J, Powell R, Cherian PT, Bowling JJ, Stephan CC, Lee RE, Hurdle JG. 2024. Chemical genetic analysis of enoxolone inhibition of Clostridioides difficile toxin production reveals adenine deaminase and ATP synthase as antivirulence targets. J Biol Chem 300:107839. doi:10.1016/j.jbc.2024.107839 39343002 PMC11566853

[14] Lau WYV, Taylor PK, Brinkman FSL, Lee AHY. 2023. Pathogen-associated gene discovery workflows for novel antivirulence therapeutic development. EBioMedicine 88:104429. doi:10.1016/j.ebiom.2022.104429 36628845 PMC9843249

[15] Liao C, Huang X, Wang Q, Yao D, Lu W. 2022. Virulence factors of Pseudomonas aeruginosa and antivirulence strategies to combat its drug resistance. Front Cell Infect Microbiol 12:926758. doi:10.3389/fcimb.2022.926758 35873152 PMC9299443

[16] Song Y, Liu C-I, Lin F-Y, No JH, Hensler M, Liu Y-L, Jeng W-Y, Low J, Liu GY, Nizet V, Wang AH-J, Oldfield E. 2009. Inhibition of staphyloxanthin virulence factor biosynthesis in Staphylococcus aureus: in vitro, in vivo, and crystallographic results. J Med Chem 52:3869–3880. doi:10.1021/jm9001764 19456099 PMC2753857

[17] Martin-Verstraete I, Peltier J, Dupuy B. 2016. The regulatory networks that control Clostridium difficile toxin synthesis. Toxins (Basel) 8:153. doi:10.3390/toxins8050153 27187475 PMC4885068

[18] Antunes A, Camiade E, Monot M, Courtois E, Barbut F, Sernova NV, Rodionov DA, Martin-Verstraete I, Dupuy B. 2012. Global transcriptional control by glucose and carbon regulator CcpA in Clostridium difficile. Nucleic Acids Res 40:10701–10718. doi:10.1093/nar/gks864 22989714 PMC3510511

[19] Antunes A, Martin-Verstraete I, Dupuy B. 2011. CcpA-mediated repression of Clostridium difficile toxin gene expression. Mol Microbiol 79:882–899. doi:10.1111/j.1365-2958.2010.07495.x 21299645

[20] Dupuy B, Sonenshein AL. 1998. Regulated transcription of Clostridium difficile toxin genes. Mol Microbiol 27:107–120. doi:10.1046/j.1365-2958.1998.00663.x 9466260

[21] Dineen SS, Villapakkam AC, Nordman JT, Sonenshein AL. 2007. Repression of Clostridium difficile toxin gene expression by CodY. Mol Microbiol 66:206–219. doi:10.1111/j.1365-2958.2007.05906.x 17725558

[22] Dubois T, Dancer-Thibonnier M, Monot M, Hamiot A, Bouillaut L, Soutourina O, Martin-Verstraete I, Dupuy B. 2016. Control of Clostridium difficile physiopathology in response to cysteine availability. Infect Immun 84:2389–2405. doi:10.1128/IAI.00121-16 27297391 PMC4962627

[23] Thanissery R, Zeng D, Doyle RG, Theriot CM. 2018. A small molecule-screening pipeline to evaluate the therapeutic potential of 2-aminoimidazole molecules against Clostridium difficile. Front Microbiol 9:1206. doi:10.3389/fmicb.2018.01206 29928268 PMC5997789

[24] Oliveira Paiva AM, Friggen AH, Hossein-Javaheri S, Smits WK. 2016. The signal sequence of the abundant extracellular metalloprotease PPEP-1 can be used to secrete synthetic reporter proteins in Clostridium difficile. ACS Synth Biol 5:1376–1382. doi:10.1021/acssynbio.6b00104 27333161

[25] Stabler RA, Gerding DN, Songer JG, Drudy D, Brazier JS, Trinh HT, Witney AA, Hinds J, Wren BW. 2006. Comparative phylogenomics of Clostridium difficile reveals clade specificity and microevolution of hypervirulent strains. J Bacteriol 188:7297–7305. doi:10.1128/JB.00664-06 17015669 PMC1636221

[26] Wu X, Cherian PT, Lee RE, Hurdle JG. 2013. The membrane as a target for controlling hypervirulent Clostridium difficile infections. J Antimicrob Chemother 68:806–815. doi:10.1093/jac/dks493 23264511 PMC3695663

[27] Iversen PW, Beck B, Chen YF, Dere W, Devanarayan V, Eastwood BJ, Farmen MW, Iturria SJ, Montrose C, Moore RA, Weidner JR, Sittampalam GS. 2004. HTS assay validation. In Assay guidance manual. Bethesda (MD).

[28] Zhang XD. 2011. Illustration of SSMD, z score, SSMD*, z* score, and t statistic for hit selection in RNAi high-throughput screens. J Biomol Screen 16:775–785. doi:10.1177/1087057111405851 21515799

[29] Ransom EM, Kaus GM, Tran PM, Ellermeier CD, Weiss DS. 2018. Multiple factors contribute to bimodal toxin gene expression in Clostridioides (Clostridium) difficile. Mol Microbiol 110:533–549. doi:10.1111/mmi.14107 30125399 PMC6446242

[30] Castro-Córdova P, Mora-Uribe P, Reyes-Ramírez R, Cofré-Araneda G, Orozco-Aguilar J, Brito-Silva C, Mendoza-León MJ, Kuehne SA, Minton NP, Pizarro-Guajardo M, Paredes-Sabja D. 2021. Entry of spores into intestinal epithelial cells contributes to recurrence of Clostridioides difficile infection. Nat Commun 12:1140. doi:10.1038/s41467-021-21355-5 33602902 PMC7893008

[31] Hofmann JD, Biedendieck R, Michel AM, Schomburg D, Jahn D, Neumann-Schaal M. 2021. Influence of L-lactate and low glucose concentrations on the metabolism and the toxin formation of Clostridioides difficile. PLoS One 16:e0244988. doi:10.1371/journal.pone.0244988 33411772 PMC7790285

[32] Fagan RP, Fairweather NF. 2011. Clostridium difficile has two parallel and essential Sec secretion systems. J Biol Chem 286:27483–27493. doi:10.1074/jbc.M111.263889 21659510 PMC3149341

[33] Torres J, Camorlinga-Ponce M, Muñoz O. 1992. Sensitivity in culture of epithelial cells from rhesus monkey kidney and human colon carcinoma to toxins A and B from Clostridium difficile. Toxicon 30:419–426. doi:10.1016/0041-0101(92)90538-g 1626323

