# Supplementary material for: Development of a high-throughput screening platform for C. difficile toxin synthesis inhibitors unveils meclizine as an antivirulence agent
Source: Antimicrob Agents Chemother. 2025 Dec 17;70(2):e00960-25. doi: 10.1128/aac.00960-25 (PMC12888876; doi:10.1128/aac.00960-25)
Supplement: Supplemental figures — Figures S1 to S4. [file aac.00960-25-s0001.pdf]

Supplementary Material for

Development of a High-Throughput Screening Platform for *C. difficile* Toxin Synthesis Inhibitors Unveils Meclizine as an Antivirulence Agent

Ravi K.R. Marreddy[1], Nghi Nguyen[2], Chetna Dureja[1], Ann-Marie McKelvey[1], Reid Powell[2], Abiola O. Olaitan[1*], Clifford Stephan[2], Julian G. Hurdle[1#]

[1]Center for Infectious and Inflammatory Diseases, Institute of Biosciences and Technology, Texas A&M Health Science Center, 2121 West Holcombe Blvd, Houston, Texas 77030, USA

[2]Center for Translational Cancer Research, Institute of Biosciences and Technology, Texas A&M Health Science Center, 2121 West Holcombe Blvd, Houston, Texas 77030, USA

*Present address: Department of Biology, University of Waterloo, 200 University Ave W, Waterloo, ON N2L 3G1, Canada

#Corresponding author email: jhurdle@tamu.edu

Index of Supplementary Information	
**Supplementary Figure 1**	Analysis of the uniformity of luminescence signals in 384-well plates.
**Supplementary Figure 2**	Dose-responses of R20291[*PtcdA::secNluc*] to meclizine (A) and various screening hits (B to D)
**Supplementary Figure 3**	Dose-responses of various strains from different ribotypes to meclizine
**Supplementary Figure 4**	Validation of transcriptional response for R20291 to meclizine
**The below are separately uploaded supplementary information**	
**Supplementary Table 1**	Significantly transcribed genes in *C. difficile* R20291 in response to meclizine compared to untreated controls.
**Supplementary Table 2**	Screening results for Prestwick chemical library

Uniformity plate 1

KEY		1	2	3	4	5	6	7	8	9	10	11	12
DMSO	A	3586216	189419	3417053	303732	3143115	167672	3048958	280047	2988819	169124	2851692	304435
FUS	B	247440	3137877	380249	2278836	240512	2474538	304013	2080580	225594	1876347	357772	1959122
VAN	C	3615097	349805	3078241	198567	3217306	337206	2450055	172407	2416202	350570	2514129	190802
GLU	D	363265	3722564	236464	2719316	322966	2414577	183138	1951599	371617	2059925	262426	2987217
	E	3597743	250053	3215929	353249	3005180	204754	2409677	310260	1766758	176820	2693130	383255
	F	266673	3530734	462903	2372350	353784	2100493	323529	2184197	216064	2164149	401202	2135005
	G	3676809	391391	3477608	218954	3215898	376699	2865296	187784	2859077	329946	2395726	205282
	H	400210	3590621	291948	3610018	384144	2727897	215102	2335907	428431	2864415	259220	2587537
	I	3659229	244521	3502377	407405	3439951	223216	2983439	338572	3224836	232438	3037919	395917
	J	228733	3326388	427834	3307182	254305	2926648	363621	2742485	300282	3007664	464854	3321541
	K	3610379	392333	3316486	239972	3209970	406209	3222631	213398	3281129	420906	3140443	244964
	L	392638	3345970	294034	3261961	433903	3102908	224483	2570773	515260	3239629	332397	3106990
	M	3621133	254352	3245074	460177	3353597	274163	3080803	414137	2915172	252335	3097683	459358
	N	315936	3501632	585635	3283141	507105	3086254	432767	2837708	378382	3093420	527495	3295292
	o	3301115	403285	3465904	232977	3154768	425157	3430185	225194	3243905	452983	2951052	234974
	P	368119	3382587	259875	3537156	421715	3501986	209220	3047341	452157	3389837	299357	3632416

	13	14	15	16	17	18	19	20	21	22	23	24
A	2394519	165181	2807063	308000	2827762	162463	2661064	330740	2750022	178021	2912713	260755
B	214087	2275466	365022	2870309	203183	1956903	342684	2616365	211591	2225261	343536	1882890
C	2272769	322713	2729642	174833	2032975	337322	1966119	185245	2491141	350805	1844149	289393
D	402092	1865447	221016	2094626	413063	2045150	184626	1902732	316947	2024857	215379	2267800
E	2606914	193955	2266386	289575	2193931	181346	2261110	293537	2156362	189210	3006306	326837
F	318783	2287300	371806	1510828	218013	1786644	347076	2300949	227084	2346254	407190	2142791
G	2829923	365198	2378130	172188	2286842	352339	2215921	191982	2666352	413869	3096913	254493
H	443512	2888105	231357	2288748	414162	2922660	215205	1990628	424441	2987247	252591	2409352
I	2876738	237283	3021011	350914	2803052	218206	2711263	370074	2567439	234141	3438342	350777
J	316951	2524625	373369	2461296	258244	2056826	409148	2600369	267277	2161114	434469	2378930
K	3480185	418697	2220925	203297	2729930	387716	2327898	214443	2723668	407440	3183190	304942
L	478515	2910182	281285	2375950	434971	2543906	261985	2719814	450818	2740558	306724	2669771
M	2889205	266379	2815517	380030	2399924	237583	2953340	419022	2476506	252865	3594940	382686
N	468811	2466842	502671	2230459	394713	2604589	536302	2592234	358721	2624147	540915	2334627
o	3226452	424333	2729071	209269	3017381	410362	2539255	220224	3021922	435295	2993707	2607678
P	473573	3462696	319776	3047724	472028	3267461	317001	3293855	470528	3153466	318409	2330834

Uniformity plate 2

KEY		1	2	3	4	5	6	7	8	9	10	11	12
FUS	A	130273	2915936	234894	2278819	120724	1708183	175569	2085702	123840	1687730	170961	1908353
DMSO	B	3284183	201994	2218652	242456	2417098	158957	2522299	228225	2251100	134676	1648646	239498
GLU	C	261968	2815067	177542	2850376	301948	1965595	156076	1814772	243955	1929188	152246	2324470
VAN	D	3565334	267445	2876397	207961	3296929	297086	3172573	192486	2431167	225616	2392820	206826
	E	204439	3362487	316905	2986040	212383	2393731	280226	2151968	206453	2305417	293514	2390809
	F	3776176	287045	3076921	375438	3230814	342376	3070812	375151	3075702	339961	2293470	397312
	G	299131	2949645	196928	3072629	307081	2586303	176525	2463102	291602	2622059	159465	1794827
	H	3329523	323010	3298740	198461	3166875	306174	3366423	196136	3090494	300373	2513920	173742
	I	199416	3386720	304054	3099948	195675	2502668	273729	2739671	182392	2459057	289690	2606626
	J	3663304	215978	3000176	306038	3258417	197768	3006885	334062	3187139	185962	2701284	265903
	K	321398	2961423	198343	3236867	330766	3184215	191819	3060769	335284	3249039	189439	2243774
	L	3305631	347698	3275769	218982	2946671	347364	3577554	246943	2944622	387232	3384001	248018
	M	198334	3380529	343814	2933253	213290	3049968	354079	3378642	222353	2944134	390049	2968271
	N	3631523	257580	3333499	388715	3474971	344797	3068577	433626	3215019	389551	3030071	445675
	o	361111	3212842	215202	3199583	388866	3308519	219386	3416089	394705	3309429	221164	2881186
	P	3344230	367742	3662540	275289	3487622	419715	3539343	361228	3468280	453311	4103768	386507

	13	14	15	16	17	18	19	20	21	22	23	24
A	133173	2903288	212335	2600271	137168	2383496	209822	2484741	134434	2388050	242566	2209283
B	1734563	179301	2257183	279307	2203915	196701	1526539	243506	1918131	208396	2063115	254676
C	282752	2611980	165986	2634970	234832	2207631	144509	2083864	230457	1957589	170156	3154249
D	2823078	295802	2071128	185290	2482742	464363	2623476	164841	2527452	327007	2103939	210847
E	205940	3149452	271033	3078189	185200	2733562	255918	2054088	192136	2567634	269663	3187808
F	2074002	200394	3055960	333883	2895060	258419	1718842	269123	2758560	260617	2561166	292521
G	269373	2931598	238421	2576840	347048	2299497	209555	2247839	356944	2502151	214225	2988585
H	2549046	333797	2934804	316509	2328938	675698	2005690	276672	2453504	362475	2507695	203672
I	187287	3261369	294867	3115485	193083	3368291	314942	2843606	183851	2527478	276188	2912587
J	3009071	207077	3108699	359489	3023964	304927	2327576	317651	3018168	260359	2848723	288910
K	338492	2978281	203247	3177573	344254	2982893	245067	2203715	317444	2725619	205892	3220081
L	2742790	346730	3455123	311264	3041962	512545	2739367	325750	3046677	387424	2496808	194429
M	219680	3238627	354474	3282855	232779	3105282	360063	2730234	225040	2589593	342222	3453832
N	3098573	225901	3313910	425704	3089610	379092	3065069	383539	3124014	365536	3155195	364572
o	372106	3069280	197404	3097639	401118	3215058	217095	3138155	427604	2939321	232612	3638119
P	3676989	388543	3739225	297380	3439446	625547	3524661	392689	3775357	470668	3775763	201513

Figure S1. Analysis of the uniformity of luminescence signals in 384-well plates. Negative controls were DMSO and vancomycin (VAN), while positive controls were glucose (GLU) and fusidic acid (FUS). The Z-factor and strictly standardized mean difference (SSMD) for different combinations of the negative and positive controls were as follows. **Plate 1**, following outlier analysis of plate 1 in Graphpad Prism 10.4.1, three outliers from the fusidic acid group were identified and removed, resulting in scores of Z'=0.26 to 0.37 and SSMD= 4.52 to 4.91; prior to removal scores were Z'=0.07 to 0.31 and SSMD= 4.32 to 4.91. **Plate 2**, Z'=0.26 to 0.37 and SSMD= 4.63 to 5.33.

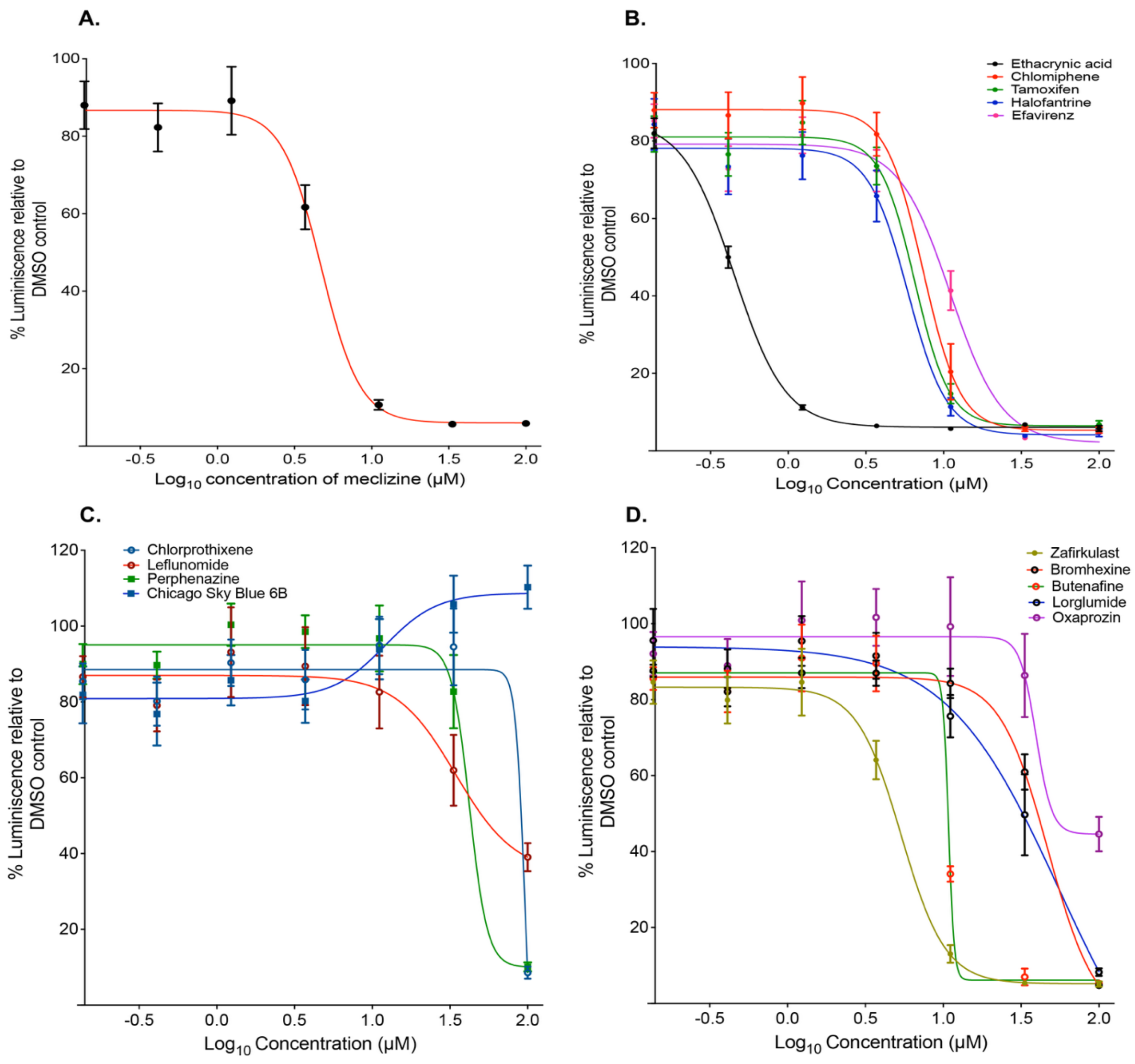

Figure S2. Dose-responses of R20291[*PtcdA::secNluc*] to meclizine (A) and various screening hits (B to D). Logarithmic cultures (OD_{600}nm ~ 0.3) were treated with 2-fold concentrations of meclizine (A) or the listed various screening hits (B). Data is from 4-6 biological replicates. Calculated EC_{50} values are in Table 1.

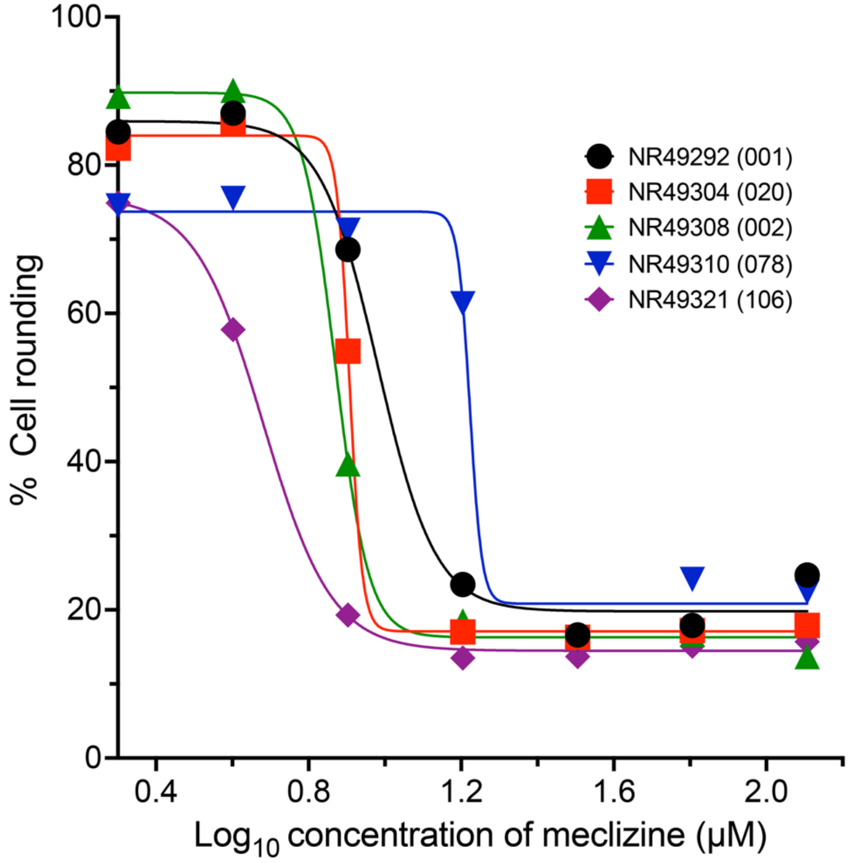

Figure S3. Dose-responses of various strains from different ribotypes to meclizine. Logarithmic cultures (OD_{600}nm ~ 0.3) were treated with concentrations of meclizine and toxins levels measured in the cell rounding assay. The names and ribotypes of the strains are shown.

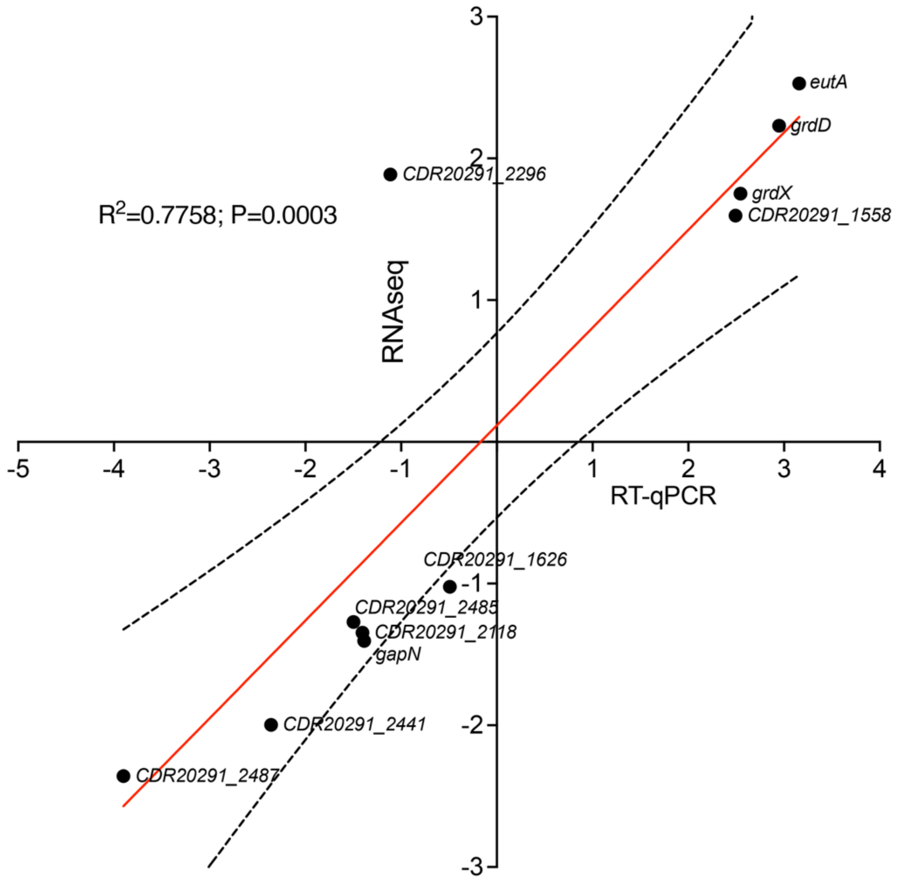

Figure S4. Validation of transcriptional response for R20291 to meclizine. Logarithmic cultures (OD_{600}nm ~ 0.3) were treated with meclizine (8 μM) for 1 hour. Pearson correlation plot of 11 genes (4 – upregulated and 7 – downregulated) analyzed by RNA-seq and RT-qPCR; Pearson's correlation coefficient R-squared=0.78, p=0.0003) in Graphpad prism 10.4.1.
