# Supplementary material for: Development of a high-throughput screening platform for C. difficile toxin synthesis inhibitors unveils meclizine as an antivirulence agent
Source: Antimicrob Agents Chemother. 2025 Dec 17;70(2):e00960-25. doi: 10.1128/aac.00960-25 (PMC12888876; doi:10.1128/aac.00960-25)
Supplement: Table S1 — Screening results for Prestwick chemical library. [file aac.00960-25-s0002.pdf]

Table S1. Screening results for Prestwick chemical library. SSMD values are below the table.

Compound	Screening concentration 10 µM		Screening concentration 100 µM	
	Percent Activity of Luminescence After Normalization by OD600			
	Replicate 1	Replicate 2	Replicate 1	Replicate 2
Azaguanine-8	43.67187149	60.3282986	52.44610563	60.86723953
Metronidazole	29.49084826	19.61442914	0	0
Allantoin	74.65005653	56.47561991	162.7795718	145.3913443
Fulvestrant	67.69576857	64.00893243	134.1383506	152.7225638
Acetazolamide	73.52555641	61.1615626	130.7269529	159.1652694
Edrophonium chloride	68.3957299	73.89674565	137.2217497	155.9259392
Metformin hydrochloride	77.36627869	75.32381691	121.3227146	110.057031
Moroxidine hydrochloride	68.99150416	67.7891274	121.3649326	0
Atracurium besylate	67.27691953	41.91261873	75.27003029	50.38870377
Baclofen (R,S)	49.66284422	83.88771704	112.9515084	114.6227095
Isoflupredone acetate	70.77646123	70.81656008	92.24736908	118.1527335
Acyclovir	75.55234297	64.64845074	123.6255063	115.1732919
Amiloride hydrochloride dihydra	85.44763611	83.22646975	137.153642	128.4309947
Diazoxide	73.01558639	62.95667823	130.7543295	104.2967628
Amprolium hydrochloride	66.81402071	89.02022826	126.9857498	116.0571681
Amidopyrine	61.79539793	93.91405198	135.6932803	130.783377
Hydrochlorothiazide	87.9415803	69.60766347	106.943893	115.7793808
Busulfan	89.14551968	73.90859348	89.61585671	84.77871969
Sulfaguanidine	58.59245815	75.63464301	131.3673289	111.1983098
Pindolol	81.21833647	59.62041671	128.0873596	135.8153654
Isoniazid	70.6482777	76.31708076	188.9133495	137.5379253
Mexiletine hydrochloride	96.11094872	66.70611799	208.7004815	131.5319405
Pentylenetetrazole	72.09807165	77.0264535	261.4309937	136.7541635
Flavoxate hydrochloride	76.19066575	100.3912626	58.2720052	42.10921016
Chlorzoxazone	57.25625563	57.99033667	127.3833864	97.68739306
Bufexamac	59.03121172	61.28061977	0	13.99783783
Ornidazole	32.21710639	27.77658818	0	49.32475123
Glutethimide, para-amino	81.0987358	78.30843949	129.4120093	114.0297667
Ethosuximide	57.69315795	59.87413499	147.8785712	90.12150037
Dropropizine (R,S)	96.7915039	65.88923733	157.0766916	104.8332847

Mafenide hydrochloride	65.81300708	59.42055998	82.77230401	122.4030097
Pinacidil	73.96821746	61.56601136	138.5338249	107.3879476
Riluzole hydrochloride	80.23020428	63.61638211	0	65.37881775
Albendazole	74.03690548	57.01593212	135.539876	86.58579701
Nitrofurantoin	53.37278152	57.4717874	0	31.23701366
Clonidine hydrochloride	81.94115946	80.7140088	235.5564551	99.36892138
Hydralazine hydrochloride	83.36945865	50.50824965	98.37189707	88.39967306
Bupropion hydrochloride	74.58431378	70.10095841	165.966919	202.9416437
Phenelzine sulfate	92.4772082	80.67526558	151.1064239	112.4403605
Alprenolol hydrochloride	77.9955407	63.79341279	112.9057525	116.0979343
Meticrane	110.1157702	92.35080075	231.9875456	103.0118871
Khellin	96.69204704	81.83895274	229.2602792	90.4885063
Benzonatate	68.2579242	80.48110975	193.6464111	123.5142214
Zimelidine dihydrochloride monc	91.2367653	82.41440939	167.2324197	99.38921403
Hydroflumethiazide	70.00261056	65.73149291	183.9312731	129.190522
Azacyclonol	66.44584824	65.70976414	179.4606265	88.10251209
Sulfacetamide sodic hydrate	60.75676101	69.9515553	223.7590733	102.4852887
Azathioprine	75.87105494	88.23247085	68.89636243	112.6584307
Heptaminol hydrochloride	62.6203779	51.96052788	177.9347386	104.0638863
Lynestrenol	39.99533302	70.37645119	0	0
Sulfathiazole	62.21409534	69.18169425	145.1586786	118.9683724
Guanabenz acetate	67.45544149	50.91584954	181.0790913	94.86944802
Levodopa	59.79474598	66.86590128	203.7596145	138.4979951
Disulfiram	53.87257064	39.77152993	0	0
Idoxuridine	65.92201675	100.9013458	209.6677017	115.440438
Acetylsalicylsalicylic acid	83.47926896	87.02512335	144.5064522	96.81845787
Captopril	66.01301347	50.9923889	198.6500223	104.8963591
Mianserine hydrochloride	60.64811224	70.50072758	127.1184462	104.7248464
Minoxidil	62.6170394	69.2273552	191.230504	91.24262253
Nocodazole	80.89025172	62.12889343	188.6613819	112.0552721
Tranexamic acid	100.2033632	87.64708328	215.3881327	145.3910577
Chlorothiazide	81.37750307	71.21754714	244.1893304	118.4757126
Etofylline	91.25650951	90.10910653	257.8234748	161.8914588
Diphenidol hydrochloride	115.8170124	113.9812453	205.5926548	124.2036212

Tranylcypromine hydrochloride	79.33741542	55.40447982	215.8978204	105.6288605
Norethindrone	95.67718908	70.56610803	261.8767314	85.45130404
Alverine citrate salt	61.42019493	60.77755772	123.2174103	122.0539892
Nortriptyline hydrochloride	81.01720676	70.16985104	72.14182047	84.48865677
Aceclofenac	51.97011442	59.08779857	118.2359521	65.88363635
Niflumic acid	54.48751712	58.00069206	42.66067202	35.55578921
Iproniazide phosphate	51.17033655	69.26698614	211.4543725	131.8066574
Isotretinoin	61.32879251	48.29998494	49.41908462	39.53540908
Sulfamethoxazole	75.12935864	53.43246774	197.0523926	131.0359428
Retinoic acid	63.34136783	50.42996287	57.22189165	59.86793889
Mephenesin	71.78972522	83.9145568	216.8908503	119.975
Antazoline hydrochloride	97.77934314	57.53530063	151.931358	73.97660185
Phenformin hydrochloride	66.32955765	47.27400632	195.6121517	100.9815406
Ethacrynic acid	4.616266174	4.370015861	0	0
Flutamide	71.86781773	67.47703581	172.9221772	87.47675681
Praziquantel	77.74143615	63.40965149	116.536782	88.64387262
Sulfaphenazole	101.0589662	90.17617941	208.6065076	123.8021963
R(-) Apomorphine hydrochloride	113.9307076	55.78562052	223.2020781	111.1726986
Panthenol (D)	96.51758219	69.47439109	229.6418622	180.685181
Amoxapine	112.853976	67.09660768	182.5629229	135.3571451
Sulfadiazine	77.231092	70.74933624	238.3198671	173.7397675
Cyproheptadine hydrochloride	86.16274439	76.26120023	109.0053318	83.96555866
Norethynodrel	65.15154371	92.971492	112.33305	88.63120613
Famotidine	94.92300515	83.15855911	218.1222805	162.4009983
Thiamphenicol	55.20724757	69.45566842	69.82731086	76.56402427
Danazol	56.27319982	77.0039835	82.61477467	95.49669778
Cimetidine	70.71600217	67.1543649	248.6809109	147.9781317
Nicorandil	74.54722584	73.47373339	156.5187511	133.0121994
Doxylamine succinate	82.93822611	67.56220954	194.3116747	137.8324107
Pioglitazone	89.57102547	69.97191491	241.0586023	134.0384337
Ethambutol dihydrochloride	84.4615712	82.54407839	189.169878	127.7926907
Nomifensine maleate	81.65795646	77.31593658	209.5259147	94.62407863
Antipyrine	79.82231365	77.78601126	197.8146565	130.903838
Dizocilpine maleate	84.07161689	79.29979374	208.6117733	141.7054987

Antipyrine, 4-hydroxy	85.45891518	129.5073114	193.7689562	131.1522564
Oxandrolone	97.14791016	96.80405871	297.7738248	124.1953701
Ampyrone	114.2157791	69.05600742	209.8682001	152.0234193
Ethisterone	113.0949906	72.10076677	258.3940359	189.5741343
Levamisole hydrochloride	100.0628056	71.18161103	265.2443784	169.3676584
Triprolidine hydrochloride	128.6560126	86.67346879	206.5122822	141.5566353
Pargyline hydrochloride	103.593351	59.66689872	195.9375495	153.0898241
Doxepin hydrochloride	131.4572201	72.35827517	156.7892698	139.8776997
Methocarbamol	75.14034194	58.19206366	209.3364071	142.5016289
Dyclonine hydrochloride	87.01439009	62.22429776	121.8491838	33.7735414
Aztreonam	75.38628469	63.9395757	180.1923575	111.1709391
Dimenhydrinate	63.94671084	59.91359217	162.7483735	113.2872055
Cloxacillin sodium salt	52.21484799	65.90907488	0	72.81110561
Disopyramide	98.68053567	72.82072106	167.995746	106.8006345
Catharanthine	72.21462088	45.1355839	250.9068511	133.7233513
Clotrimazole	25.53413212	45.09517955	0	0
Pentolinium bitartrate	78.44231512	63.89019721	203.3175768	115.8586894
Vinpocetine	93.5189033	65.88147257	38.37398004	81.98165741
Aminopurine, 6-benzyl	66.73412867	59.15569314	60.90223883	38.61905803
Clomipramine hydrochloride	66.19647008	101.548748	50.4931909	41.55116522
Tolbutamide	87.91946883	79.52986733	214.5485406	115.3263517
Fendiline hydrochloride	17.55437532	56.04977626	0	0
Chloramphenicol	95.78162468	77.21143163	0	67.01724518
Naloxone hydrochloride	121.9896719	76.04564712	215.3018354	164.6236179
Epirizole	77.65701903	76.47816862	212.2072896	187.2209192
Metolazone	101.6364123	70.38044192	141.7247977	90.07538466
Diprophylline	120.2458367	57.86994288	210.6689723	170.6999426
Ciprofloxacin hydrochloride mor	103.7721859	48.49602213	101.6541984	84.48643231
Triamterene	100.7375272	69.41043456	161.2421812	80.37331997
Ampicillin trihydrate	92.50717216	79.66924194	0	144.2594533
Dapsone	102.2669621	51.81158762	199.8498434	103.2925419
Haloperidol	88.41814023	72.61962521	103.8730066	100.680691
Troleandomycin	82.76335132	78.78203806	186.4881121	103.3851634
Naltrexone hydrochloride dihydr	98.21053505	61.38392826	195.8294119	81.70741636

Pyrimethamine	116.4401244	54.78303589	241.7289633	113.7582796
Chlorpheniramine maleate	106.2601718	47.81258542	150.2652158	97.04138464
Hexamethonium dibromide dihy	119.8801935	84.37996804	167.6957446	100.4675476
Nalbuphine hydrochloride	97.37893168	66.75289811	187.9631526	129.4756022
Diflunisal	64.53856527	33.4184168	34.40767705	13.73942367
Picotamide monohydrate	105.9785968	67.75235488	136.1974887	123.463509
Niclosamide	87.73170145	52.29730543	0	130.2226334
Triamcinolone	100.5065727	74.34640508	177.8420273	97.08885794
Midodrine hydrochloride	117.7609759	115.8498411	234.1928584	147.1275161
Vincamine	116.4374091	73.76578096	249.8775733	171.7815113
Thalidomide	110.8203368	66.22185268	219.5696335	169.8664414
Indomethacin	118.8189805	84.10051331	84.91712976	119.552954
Oxolinic acid	120.6060516	50.54552613	166.4190409	136.0588738
Cortisone	120.2049868	76.69073795	197.8769729	125.4115408
Nimesulide	105.9447061	34.84554677	158.9752988	110.1305145
Prednisolone	107.7008346	67.1608664	281.7876034	98.88993249
Asenapine maleate	107.5234763	69.54654989	176.6608353	99.36860402
Fenofibrate	76.14611885	87.67402709	182.3318248	87.21926932
Pentoxifylline	94.02820994	97.7551855	228.6282546	113.5874051
Bumetanide	109.2710594	56.15461949	159.3684202	90.86080991
Metaraminol bitartrate	98.98046437	52.99855575	222.0624571	103.0511702
Labetalol hydrochloride	112.9726109	74.63315174	203.2487488	121.1547033
Salbutamol	101.3792788	78.47794815	238.1530887	100.2495265
Cinnarizine	112.7748658	46.96576664	75.41671414	54.71422131
Prilocaine hydrochloride	122.0891072	75.25464024	189.5772149	111.0075067
Methylprednisolone, 6-alpha	106.8810063	119.392343	217.8231523	155.1830426
Camptothecine (S,+)	98.55039399	51.51957998	215.4773347	108.6661006
Quinidine hydrochloride monohy	142.356674	54.04937771	173.8862037	102.2533693
Procaine hydrochloride	132.1087944	79.1013528	228.5825708	180.9031717
Bromocryptine mesylate	40.62251064	36.78798942	110.5100891	37.80826854
Moxisylyte hydrochoride	114.9324625	63.946326	244.2687029	159.5135148
Amfepramone hydrochloride	134.2469245	75.5841391	225.50254	102.5757374
Betazole hydrochloride	118.3861915	74.57153746	229.3577565	155.665642
Dehydrocholic acid	123.323839	65.53254253	246.8745834	89.87998246

Isoxicam	109.8478121	90.33812988	64.97883984	74.59107603
Tioconazole	0	0	0	0
Naproxen	96.72315843	61.47228571	140.6440864	67.33192324
Perphenazine	104.0497269	77.43442724	30.44081642	30.48377418
Naphazoline hydrochloride	122.8317943	66.20451247	178.1898773	74.09408731
Mefloquine hydrochloride	73.56104442	92.76291286	0	0
Ticlopidine hydrochloride	108.4817228	62.50647842	163.1422896	63.49048891
Isoconazole	0	0	0	0
Dicyclomine hydrochloride	116.8772974	76.37307626	80.35101034	75.35867508
Spironolactone	123.0148035	87.6000903	138.6655428	113.5528382
Amyleine hydrochloride	136.0514267	47.73136455	193.9584724	88.11090631
Pirenzepine dihydrochloride	131.1342091	68.5360602	190.2552116	97.83828187
Lidocaine hydrochloride	125.3931415	66.02669719	161.0433849	115.4728673
Dexamethasone acetate	139.1829796	35.41131126	291.7561963	93.81024881
Ranitidine hydrochloride	133.9431177	95.34747681	225.1954993	137.2125473
Fludrocortisone acetate	127.5749236	83.05477362	321.3074336	170.4493572
Tiratricol, 3,3',5-triiodothyroacet	14.45730633	41.54013447	0	0
Fenoterol hydrobromide	135.0338613	93.04609111	234.7523985	132.0953829
Flufenamic acid	62.68262602	49.70367317	12.53156897	0
Homochlorcyclizine dihydrochlo	112.1819042	69.39115388	62.3263937	70.3553147
Flumequine	105.407894	75.90361738	98.02149055	90.08598598
Diethylcarbamazine citrate	124.0173562	81.38844463	162.8038999	94.64526728
Tolfenamic acid	45.79841061	70.06215843	5.246295091	7.888914945
Chenodiol	102.7291348	63.95154178	54.26107263	28.45058789
Meclofenamic acid sodium salt	35.19671349	53.78256485	7.783904983	0
Perhexiline maleate	31.51832656	56.66471608	0	0
Tibolone	115.0185683	66.74125494	168.708443	109.0368544
Oxybutynin chloride	126.2878615	65.13977698	126.6335461	69.90481752
Trimethoprim	122.9383839	71.010612	234.8877644	78.65451141
Spiperone	98.4658986	57.04898027	157.613955	106.8537657
Metoclopramide monohydrochlc	119.4196404	47.73505483	214.9916596	112.7422603
Pyrilamine maleate	117.1849567	96.62486983	177.7783978	112.2365779
Fenbendazole	114.3364182	49.94586302	171.8811034	112.1197685
Sulfinpyrazone	89.79514096	38.75706661	75.00616105	31.8883496

Trichlorfon	119.9632676	79.32461839	284.7880049	140.4629411
Glipizide	102.4433171	94.6467332	282.6734352	133.7247438
Carbamazepine	102.1036452	102.4993333	258.3689626	132.5058856
Loxapine succinate	119.6206693	100.6185821	323.4585229	101.4062979
Triflupromazine hydrochloride	83.34517162	81.15439854	44.7967145	21.25694838
Hydroxyzine dihydrochloride	93.75595594	63.55404013	103.8117181	69.22833771
Mefenamic acid	83.50216747	82.10345485	26.21281168	40.19363318
Diltiazem hydrochloride	89.83715952	96.29704366	216.7753947	207.0871914
Acetohexamide	74.03851859	59.36987369	227.7276474	78.028638
Methotrexate	62.45072685	71.09246511	35.35329971	10.59192436
Sulpiride	88.642621	99.05707573	224.4953342	90.12360258
Astemizole	107.4519776	116.0330084	0	0
Benoxinate hydrochloride	122.3308123	76.85757016	312.7703833	125.500415
Clindamycin hydrochloride	0	10.89326404	0	0
Oxethazaine	79.10068376	77.96027801	0	0
Terfenadine	76.49290808	93.49645609	0	78.51929898
Pheniramine maleate	92.7520458	45.91740806	173.74457	82.81072979
Cefotaxime sodium salt	97.86602023	52.98476444	144.2684064	100.0889891
Tolazoline hydrochloride	89.05720889	53.73572797	213.9641132	104.7051458
Tetracycline hydrochloride	0	0	0	0
Piroxicam	88.45845033	74.71718029	94.26384724	80.18273701
Dantrolene sodium salt	86.21631402	100.0979088	173.1129934	113.2859055
Pyrantel tartrate	111.2809069	90.41240196	248.5931283	96.21652503
Trazodone hydrochloride	121.8143965	102.1900332	191.091196	111.6349647
Fenspiride hydrochloride	104.0848713	58.34544222	238.435381	109.7712936
Glafenine hydrochloride	98.55281986	83.16087199	172.8675581	69.62944705
Gemfibrozil	66.93304753	77.80742641	44.46837402	24.48102749
Pimethixene maleate	69.06962579	69.39915355	48.33664366	5.389662245
Mefexamide hydrochloride	79.3007882	81.02958654	260.6470323	88.58849126
Pergolide mesylate	72.83694935	59.73151494	137.4335263	94.25612553
Tiapride hydrochloride	95.64231999	101.593763	223.6844827	94.07881212
Acemetacin	87.57470146	58.5315761	61.22822957	47.12793607
Mebendazole	80.96387151	83.41375784	238.8361994	89.79607377
Benzydamine hydrochloride	125.1782503	55.73538631	214.4950746	82.71647631

Fenbufen	66.16062588	87.2208262	269.2844899	110.5537979
Fipexide hydrochloride	90.47778871	54.94590898	212.043151	77.10242782
Ketoprofen	106.9004434	48.90615384	226.2821636	100.6831251
Mifepristone	80.14063017	89.26104294	117.0845703	41.5591367
Indapamide	85.64474133	47.93583595	256.6134157	94.54129924
Diperodon hydrochloride	127.7916958	51.22271415	92.79570823	0
Morantel tartrate	135.3042844	64.81014037	265.2955805	124.656427
Verapamil hydrochloride	130.0937973	72.7212048	305.6199793	142.0290864
Homatropine hydrobromide (R,S	115.6982424	84.20846825	292.1891423	124.6760509
Dipyridamole	123.0919907	73.86112474	315.4461105	49.12399165
Nifedipine	94.77654558	83.53908538	0	0
Chlorhexidine	0	10.04249897	0	0
Chlorpromazine hydrochloride	102.9654959	74.81711624	57.52617833	30.65147077
Loperamide hydrochloride	91.38347643	71.12189012	50.08819256	0
Diphenhydramine hydrochloride	107.5374657	65.32613693	234.3702411	68.72566831
Chlortetracycline hydrochloride	0	0	0	0
Minaprine dihydrochloride	103.1743271	88.05509989	252.642686	51.6692866
Tamoxifen citrate	5.127143851	9.231176611	0	0
Miconazole	53.31820428	79.60798424	0	0
Nicergoline	116.174672	64.61309831	237.043537	90.58303179
Isoxsuprine hydrochloride	131.6359833	78.94091268	185.4454376	104.3420935
Canrenoic acid potassium salt	122.9428105	86.98667626	518.4054223	86.61603298
Acebutolol hydrochloride	98.34493749	41.55969807	230.5521026	70.21551342
Thioproperazine dimesylate	101.9007503	47.18938917	85.22072082	71.28681146
Tolnaftate	88.1802314	61.03118337	117.2904571	70.32625035
Dihydroergotamine tartrate	104.6595205	42.03907268	163.8403162	88.24343841
Norfloxacin	122.8153954	86.47337523	185.5519168	90.96076378
Lisinopril	124.9861384	79.79467717	221.7017681	143.0932778
Antimycin A	98.63412007	67.43090378	159.5368669	56.53548017
Lincomycin hydrochloride	130.6341259	39.39952777	0	0
Xylometazoline hydrochloride	120.8579289	54.48808361	190.2466973	81.93739273
Telenzepine dihydrochloride	126.242268	79.62633577	234.3125339	88.13348362
Oxymetazoline hydrochloride	115.011626	65.36863858	187.283488	72.19420595
Econazole nitrate	0	0	0	0

Nifenazone	98.27634047	80.12096024	269.3359489	90.69484
Bupivacaine hydrochloride	97.0376329	64.64278538	225.4552599	49.15961988
Griseofulvin	100.1467765	91.35650777	187.6456744	56.61726484
Clemastine fumarate	91.80170878	66.98208769	53.09827533	19.0541151
Clemizole hydrochloride	112.1929125	90.22095772	0	124.2971499
Oxytetracycline dihydrate	0	11.59031968	0	3.46981429
Tropicamide	103.8546585	68.39186363	245.5126564	78.03937404
Pimozide	111.8935708	59.85775891	0	11.50556149
Nefopam hydrochloride	117.8157139	49.02275187	228.3971755	79.94961237
Amodiaquin dihydrochloride dih	101.3349396	78.29264497	146.4524997	70.82494978
Phentolamine hydrochloride	107.7264571	50.52283819	178.9440874	90.17038158
Mebeverine hydrochloride	138.1828821	85.64454557	165.5519431	73.58359971
Todralazine hydrochloride	121.7630609	78.66772658	248.6846671	130.9886291
Erythromycin	105.24853	27.70514034	218.306507	66.9223733
Imipramine hydrochloride	112.6683677	56.38470601	172.1853097	101.5409055
Chloroxine	0	0	0	0
Sulindac	89.01251325	74.24924305	145.3127097	136.4495348
Didanosine	116.2130993	69.75201705	289.1039464	94.92787368
Amitryptiline hydrochloride	113.355896	68.13724751	130.9995297	74.8742526
Josamycin	127.8657359	40.798128	0	0
Adiphenine hydrochloride	129.1494767	61.3943731	274.7580035	76.3765729
Paclitaxel	101.7297717	84.66483653	227.838407	93.19673859
Dibucaine	110.2217768	73.02936852	224.5598046	8.759333033
Ivermectin	122.1773652	85.51886536	103.9851925	47.01008998
Prednisone	115.4236215	76.64355332	222.1781577	117.7994672
Gallamine triethiodide	122.1053402	60.37634919	233.7137822	119.4630532
Thioridazine hydrochloride	38.60453113	80.38346995	0	0
Neomycin sulfate	124.8711881	85.82808311	283.2874606	116.4057902
Diphemanil methylsulfate	132.037917	47.93777682	214.4693375	84.36916492
Dihydrostreptomycin sulfate	124.0031652	58.8057175	256.1319607	119.6591921
Trimethobenzamide hydrochlori	126.2627039	63.12089217	274.8779249	110.7057915
Gentamicine sulfate	130.8619201	54.09229772	241.3882379	102.4395139
Etodolac	111.1117632	72.28765542	183.9122221	129.3885184
Ifenprodil tartrate	122.2516073	45.69170812	218.3522162	96.42973308

Scopolamin-N-oxide hydrobrom	119.3307101	67.89759058	217.0989542	85.53563976
Flunarizine dihydrochloride	0	8.4303902	0	4.041282397
Hyoscyamine (L)	117.6287499	54.94867171	248.7697217	109.2495846
Trifluoperazine dihydrochloride	66.29727651	57.74989806	0	0
Chlorphensin carbamate	112.6734412	78.76938603	228.8804099	86.96322802
Enalapril maleate	114.4926574	79.93279657	225.9573884	119.9719563
Fadrozole hydrochloride	84.43522702	88.01925144	0	100.4976915
Minocycline hydrochloride	0	0	0	0
Dilazep dihydrochloride	118.3242167	101.7885112	260.3551778	77.02461615
Glibenclamide	97.49584755	56.53554944	45.09728625	37.74640621
Ofloxacin	103.8435786	84.47112049	158.0323432	125.0005599
Guanethidine sulfate	117.8070572	68.69855081	232.4948917	94.74297068
Lomefloxacin hydrochloride	112.2810059	67.66714081	244.3693294	73.24397151
Quinacrine dihydrochloride dihy	91.48647212	50.74448449	99.69172998	29.07027516
Orphenadrine hydrochloride	126.7519775	47.01877889	205.2657181	88.85548728
Clofilium tosylate	53.38116859	45.50323946	28.45195017	0
Proglumide	126.9001791	52.11151049	252.8072958	150.7047326
Fluphenazine dihydrochloride	91.12893185	63.88585707	17.10019674	0
Streptomycin sulfate	98.88031608	61.93703583	130.3288277	59.9176245
Testosterone propionate	65.12592961	85.45494286	79.08344397	30.8856304
Alfuzosin hydrochloride	73.69569553	59.28849001	174.550344	126.5541646
Haloprogin	26.37802354	20.71311564	0	0
Chlorpropamide	75.91219064	56.96507987	126.648449	58.40914758
Thyroxine (L)	33.3005917	81.04782299	39.02788698	24.92825902
Phenylpropanolamine hydrochlo	71.70366115	80.68544753	160.284266	119.108792
Idebenone	68.54978121	58.75018452	0	57.46188566
Ascorbic acid	68.85861672	84.85319117	132.0244723	97.06190568
Pepstatin A	71.73842998	60.38724644	179.5263666	142.0758128
Methyldopa (L,-)	69.18115253	89.98816154	165.5414206	83.89959542
Morpholinoethylamino-3-benzoc	89.49755717	72.36623719	99.90220507	81.11337564
Cefoperazone dihydrate	56.92913233	66.70723067	0	0
Adamantamine fumarate	76.79979651	81.01779347	155.8377753	101.4073878
Zoxazolamine	73.70770102	84.88852984	141.0444801	89.03592223
Butoconazole nitrate	0	0	0	0

Tacrine hydrochloride	84.9013549	57.94311743	118.9080466	81.66460039
Amiodarone hydrochloride	0	0	0	0
Bisoprolol fumarate	63.05332119	62.92570664	148.7496179	147.9610255
Amphotericin B	72.72309159	59.02612943	111.118544	52.84149486
Serotonin hydrochloride	90.31035982	64.41439713	211.9396879	83.81695264
Ziprasidone Hydrochloride	67.40073406	70.58140085	69.57314771	54.25915411
Cefotiam hydrochloride	76.96699394	75.23969118	164.7116702	228.1106553
Mevastatin	88.48560557	55.50406613	75.30396895	63.87847305
Rofecoxib	77.51106179	63.95075481	150.1323362	55.73121174
Pyridostigmine iodide	77.50084887	59.42109424	187.58088	78.58891126
Benperidol	83.69106075	87.2079586	151.6687667	146.7310994
Pentobarbital	106.6815558	86.8221331	181.2676576	121.6287621
Cefaclor hydrate	93.30383041	56.47619304	244.5258223	132.6063399
Atropine sulfate monohydrate	111.1191504	65.63624894	186.5289015	113.9593924
Colistin sulfate	67.67265793	73.81252087	81.59716518	72.04047542
Eserine sulfate, physostigmine s	80.20344934	91.95066325	180.3783683	105.6443308
Daunorubicin hydrochloride	54.38144735	82.3512542	0	19.99234267
Itraconazole	77.54422878	77.7241418	171.7870849	93.83319538
Dosulepin hydrochloride	81.54544231	64.3455122	80.29546278	95.71608576
Acarbose	70.72524798	57.71696404	208.1968633	92.97286875
Ceftazidime pentahydrate	87.00256199	94.14077698	85.72742132	272.4453732
Entacapone	70.99391104	57.02931541	0	29.65854481
Iobenguane sulfate	93.14702408	51.98268749	219.3404706	105.7147164
Nicotinamide	82.65692509	68.99091238	176.0853566	141.5915127
Tremorine dihydrochloride	63.48587509	66.06536366	89.38341341	49.65462942
Androsterone	98.95495554	100.9101797	308.9283708	99.30893285
Practolol	66.82793981	88.99208152	197.889438	112.8517731
Amifostine	73.18458039	80.49177064	213.2646367	166.1966825
Zidovudine, AZT	60.60537204	73.24298989	214.9311854	96.33235215
Carbarsone	59.47682849	78.40812528	177.3979265	95.44516557
Sulfisoxazole	76.79596783	103.8755622	199.5595382	77.71610215
Amlodipine	93.96744003	72.67564918	140.8666994	83.25553522
Zaprinast	46.24690603	78.69709571	71.94754729	76.14008506
Modafinil	83.680492	70.61178265	160.6492	161.4910129

Chlormezanone	63.77165491	92.53517177	196.5221739	90.03277233
Bacampicillin hydrochloride	63.73384939	62.74399288	137.6836905	97.85188292
Procainamide hydrochloride	63.44893599	70.28401646	200.5021508	123.8844627
Lamivudine	54.18360489	74.34861495	163.4108313	94.99317864
N6-methyladenosine	71.69018821	79.90055974	196.8959747	116.817582
Biotin	76.9415962	93.64979778	188.4535567	118.2096217
Guanfacine hydrochloride	80.37695382	74.07301103	167.7321873	97.19909381
Bisacodyl	44.21297517	49.13646422	68.73043758	23.57229216
Domperidone	72.17712945	67.74729989	100.8645932	69.78121127
Erlotinib	73.35021852	55.81568906	156.0247131	68.58903742
Metixene hydrochloride	58.01334925	77.94318115	35.56490279	9.044204649
Tetracaïne hydrochloride	108.6035133	99.84312503	175.9987797	102.9181826
Nitrofural	55.08784107	67.85497784	0	57.45445589
Mometasone furoate	59.17752317	63.08449193	131.3328437	117.7015689
Omeprazole	53.3244173	63.88149569	138.5710893	82.94872106
Troglitazone	0	32.66419793	0	0
Propylthiouracil	60.46280664	75.21057518	197.6678849	113.4839721
Dacarbazine	47.74057874	74.36350653	107.6414756	97.393374
Terconazole	60.07712904	80.96441158	152.1562841	122.2622264
Tenatoprazole	50.23640792	78.34916101	99.92419718	79.91089953
Tiaprofenic acid	77.71939036	70.17525669	210.1601494	79.30117714
Acetopromazine maleate salt	55.11388473	75.3533494	109.1528632	64.2767099
Vancomycin hydrochloride	0	0	0	0
Escitalopram	71.86164377	58.12195155	130.2432227	94.42275705
Artemisinin	78.23661785	106.7994116	123.4295024	121.8651596
Ropinirole HCl	65.18793373	60.01887201	183.4273388	89.42640705
Propafenone hydrochloride	57.79093949	63.11380095	185.784306	133.1374322
Lacidipine	42.6342911	53.82471798	45.03389767	10.7625722
Ethamivan	53.76817902	38.06609172	194.0413963	136.7004754
Argatroban	84.57127651	67.05530155	192.6310163	123.4871455
Furosemide	109.5914743	89.567671	244.6684994	143.3049883
Suloctidil	116.2954607	134.4370454	0	74.48044328
Methapyrilene hydrochloride	81.60488002	95.75602411	214.835869	138.3369552
Zotepine	67.80997503	97.87114308	0	0

Desipramine hydrochloride	64.71948058	85.29877493	103.5239638	95.245785
Carisoprodol	81.00882782	70.53503516	182.0875858	96.0065651
Clorgyline hydrochloride	88.33314673	93.0418123	141.7641507	108.4621813
Cephalosporanic acid, 7-amino	84.29254274	86.82140772	209.2581361	111.141875
Clenbuterol hydrochloride	66.60931101	52.3935802	206.3891699	94.81071564
Chicago sky blue 6B	68.31809778	62.33371948	17.28441405	7.544500399
Maprotiline hydrochloride	80.08145036	77.99719328	107.9330075	65.99541831
Buflomedil hydrochloride	92.23167424	70.44259027	210.2582089	132.3508905
Thioguanosine	83.37871273	64.23178093	130.2842711	83.53123467
Dibenzepine hydrochloride	63.76094121	63.09805521	138.6000536	101.8863919
Chlorprothixene hydrochloride	76.02513111	70.67574281	23.58867684	7.642266606
Roxatidine Acetate HCl	81.59588266	99.28343472	172.1602364	92.68449336
Ritodrine hydrochloride	96.13080254	77.79541047	181.7008577	128.3008607
Valacyclovir hydrochloride	75.36688126	83.8283719	196.6524552	123.0288273
Clozapine	74.00617472	57.13253145	101.1172343	116.5054295
Cisapride	81.67561358	63.05346954	170.1248485	78.25841181
Vigabatrin	110.5083794	106.5396875	258.4822929	140.8843143
Reboxetine mesylate	121.7696229	118.2936479	208.8035279	118.5377138
Biperiden hydrochloride	107.564614	87.16965683	118.0865597	160.5855351
Camylofine chlorhydrate	83.76835754	64.6700706	199.0873953	114.5495048
Cetirizine dihydrochloride	62.32088104	71.95897641	182.6261017	87.56073502
Papaverine hydrochloride	83.17207866	63.93840107	137.3931782	73.30522174
Etifenin	61.63888987	61.51115336	192.6140341	118.6544118
Yohimbine hydrochloride	59.47678038	71.98722984	186.4757341	111.3262856
Metaproterenol sulfate, orcipren	80.68582594	81.37215718	190.0224254	103.7523892
Voriconazole	72.93087716	89.69720315	201.9548882	91.06787399
Sisomicin sulfate	58.52519834	90.91454048	184.2077518	85.50658656
Alfacalcidol	55.04963602	67.23136123	112.6701558	0
Sibutramine HCl	63.91408164	51.97208127	69.29453197	58.09232791
Cilostazol	88.00519624	61.302931	217.247059	88.59858232
Acenocoumarol	79.78866	76.16983354	124.998427	83.54025942
Galanthamine hydrobromide	87.55566984	59.0196127	201.2436323	131.3904096
Bromperidol	56.64610991	59.1617145	122.9017635	115.6378444
Azelastine HCl	86.91073708	46.74996195	122.9809893	69.79972954

Cyclizine hydrochloride	76.35103753	40.51570408	128.9080978	93.9952606
Etretinate	69.48472486	67.31738023	72.26128146	16.14956122
Chlorthalidone	133.0250365	92.9937197	226.4641659	148.2710781
Pefloxacine	112.8675341	138.134735	111.2107009	139.0285992
Dobutamine hydrochloride	74.44918412	108.2114352	186.3830067	174.3228439
Corticosterone	90.09112436	90.42380939	276.2702427	257.8706553
Moclobemide	69.97026208	117.7328769	208.168909	144.8641953
Cyanocobalamin	30.21117579	82.95069193	143.3815372	34.03246005
Clopamide	68.44350591	86.13981699	194.7696027	103.5580691
Cefadroxil	83.64219919	72.5543071	199.4862963	136.8461705
Hycanthone	75.87106992	63.87137185	90.7453678	25.95362519
Cyclosporin A	83.45492429	57.87007135	104.6116465	77.70576317
Adenosine 5'-monophosphate n	71.87455761	55.16067308	172.1999415	84.49295713
Digitoxigenin	83.28845009	58.23003147	224.196462	117.3894555
Amoxicillin	0	49.81603177	0	0
Digoxin	62.82260269	69.3578854	137.6228976	68.80069288
Cephalexin monohydrate	73.87538662	66.78194675	191.2925525	100.9477931
Doxorubicin hydrochloride	44.96939035	70.05332162	0	18.36880369
Dextromethorphan hydrobromid	85.49367147	57.31440688	136.9188108	94.40675353
Carbimazole	88.30328137	23.09093078	186.8009565	102.3631938
Droperidol	68.52862657	23.28886286	182.8500999	110.5596887
Epiandrosterone	68.86438811	40.48836933	286.4498687	90.69851289
Fluoxetine hydrochloride	108.9297703	127.5326749	79.4705582	69.33833789
Emedastine	128.9088024	136.1011629	268.0746315	157.6531686
Iohexol	116.4936533	149.3709806	255.4183465	261.602203
Etofenamate	62.74333771	50.33304085	0	0
Norcyclobenzaprine	88.38048888	136.8896969	99.24051966	133.7997746
Zaleplon	84.52014003	172.4081486	206.2750562	203.1840426
Pyrazinamide	75.27617136	53.44177152	233.3764305	115.4619378
Diclofenac sodium	76.23208646	63.664702	96.53597485	91.68616357
Trimethadione	84.62430535	69.20356603	209.0061603	89.57729957
Exemestane	78.24221918	71.76097333	143.4995203	67.97831825
Lovastatin	59.24296003	61.63518895	0	80.58383681
Fomepizole	63.90787636	64.26604549	181.6916392	103.7521733

Nystatine	60.61496985	67.43060489	143.8786756	180.4685182
Temozolomide	65.06529743	55.4270443	141.0143044	82.23807309
Budesonide	105.6431897	118.9799037	241.7040895	103.5455133
Xylazine	86.65390808	48.10243783	202.3816295	99.32153508
Imipenem	58.09753437	28.54741304	167.200059	95.47254516
Celiprolol HCl	85.21109247	23.11904968	165.9540042	79.40613091
Sulfasalazine	89.84236958	23.06189692	226.4052402	103.6540127
Zopiclone	70.20976655	38.43265351	207.0298032	88.5399047
Bambuterol hydrochloride	148.472754	105.1062201	296.6848194	167.478917
Estradiol-17 beta	129.0388018	125.8346058	323.1690982	112.091188
Betamethasone	105.5269259	106.4424923	341.1540604	208.9180349
Clobutinol hydrochloride	111.1398231	150.2287042	259.2454156	268.9832424
Colchicine	119.4356783	129.7328959	210.6671683	173.6554614
Gabazine bromide	85.38015356	91.74539213	0	0
Metergoline	135.4858015	105.7871313	78.05158201	19.15255711
Oxcarbazepine	127.3765375	80.0509774	213.3553843	141.7415499
Brinzolamide	93.08703161	100.1860539	205.0524092	61.90476395
Cyclobenzaprine hydrochloride	0	0	80.43902671	0
Ambroxol hydrochloride	90.78366332	0	186.1537682	105.251437
Carteolol hydrochloride	113.4320794	0	222.9678978	141.2221531
Benfluorex hydrochloride	130.6679485	54.86343924	197.3660443	154.8892516
Hydrocortisone base	78.67871833	71.77295186	233.1290101	93.55489461
Bepridil hydrochloride	49.34527207	59.873991	0	0
Hydroxytacrine maleate (R,S)	110.2782325	57.7467619	82.26315053	80.18668623
Meloxicam	69.21679816	36.59142101	25.7275611	62.626945
Pilocarpine nitrate	91.66257042	36.89381124	246.0428363	109.2495908
Benzbromarone	35.58754313	42.63788583	0	0
Dicloxacillin sodium salt hydrate	26.67651525	21.86579682	0	0
Lofexidine	116.3948685	122.3762046	244.0928204	122.7777638
Tranilast	28.6825175	43.59138295	11.07350713	5.141525574
Thiostrepton	116.36477	125.8142658	0	212.430358
Tizanidine HCl	128.5672392	83.30599363	269.1062681	148.5164772
Miglitol	110.7277617	135.7155496	333.7002073	145.1072248
Zafirlukast	10.56198877	23.56028858	0	0

Tiabendazole	111.3647997	96.07162176	187.9220819	95.10495388
Butenafine Hydrochloride	16.82243742	46.73475612	25.14268808	17.87814231
Rifampicin	0	0	0	0
Carbadox	65.62731288	0	0	21.40564299
Ethionamide	115.5550734	0	218.442249	156.6159722
Rimantadine Hydrochloride	109.6428248	32.09734529	192.4003585	103.0815098
Tenoxicam	86.25737189	27.06142031	65.87251468	63.93675075
Eburnamonine (-)	91.18431572	0	224.3259009	115.1150891
Triflusal	102.8671626	51.55802987	111.0134769	93.29183151
Oxibendazol	102.0454354	45.6936913	221.180582	116.6127224
Mesoridazine besylate	64.50537984	23.17019082	134.9243929	104.956056
Ipsapirone	84.19955342	27.82927523	254.315384	87.73744115
Trolox	105.0813244	30.53776603	233.5709181	0
Hydroxychloroquine sulfate	108.4456523	36.11657836	192.7234773	92.28429767
Ketotifen fumarate	130.4101891	117.7493862	184.7191258	131.278883
Alizapride HCl	117.2878721	133.7083481	280.1818761	177.0563979
Debrisoquin sulfate	73.40422305	119.7845746	220.937889	210.0167341
Stanozolol	154.6359563	126.1315037	349.5757928	314.6997686
Amethopterin (R,S)	87.27300755	102.4647534	204.2353026	60.23306981
Calcipotriene	55.36980846	113.3969717	0	0
Methylergometrine maleate	66.95531615	117.7329625	231.4113677	80.12870455
Linezolid	0	0	0	0
Methiothepin maleate	65.4669216	112.9135571	0	0
Mebhydroline 1,5-naphtalenedis	83.81317868	68.29554691	112.2252305	87.91922517
Clofazimine	67.99198544	113.0626181	0	72.91054146
Meclocycline sulfosalicylate	0	0	0	0
Nafronyl oxalate	102.0026547	0	151.7096434	101.8288204
Meclozine dihydrochloride	8.895773644	24.14060898	11.77234091	6.262700137
Bezafibrate	65.83216551	0	260.0420832	137.9811806
Melatonin	70.35385034	0	241.0039258	111.3535807
Nefazodone HCl	93.9888844	0	52.25652968	23.07828225
Butalbital	90.1041277	43.61541636	213.6978331	135.8162725
Clebopride maleate	86.66191344	29.42219233	238.3918015	135.9588699
Dinoprost trometamol	80.44840864	31.15148491	246.1576379	98.47784321

Pirenperone	113.7260583	125.804918	271.3995114	142.158758
Loracarbef	106.6318781	108.3744722	220.4575199	186.3780492
Isoquinoline, 6,7-dimethoxy-1-m	109.0682615	105.2969416	244.8569532	223.0193457
Fenipentol	115.2830232	65.70350064	245.7473766	162.0494719
Phenacetin	104.655764	103.0384097	273.827782	142.7334792
Diosmin	104.5164466	108.366031	246.8251202	137.9421804
Atovaquone	75.57219151	83.06476911	192.6619736	112.1530986
Carbidopa	105.4325562	108.5822094	0	107.3668139
Methoxamine hydrochloride	108.6922518	88.28288457	227.780862	136.1467529
Chrysene-1,4-quinone	92.9296049	88.19537777	207.7839995	95.27962463
(S)-(-)-Atenolol	52.19808695	44.9599385	187.4126172	137.8050215
Demecarium bromide	70.6189946	0	138.0565216	0
Piracetam	76.68144463	0	217.4534453	173.2232225
Quipazine dimaleate salt	112.49035	44.75032997	110.3428008	79.17316498
Phenindione	98.65391532	33.06057283	171.5619684	122.0058977
Acipimox	79.25837734	32.2232944	230.0884908	104.759219
Thiocolchicoside	71.70646085	32.51327688	239.6517899	120.6732064
Diflorasone Diacetate	98.52371194	41.96879835	284.5407299	174.1217952
Clorsulon	107.1715228	25.49005844	205.4954473	127.2930705
Acamprosate calcium	80.24469766	73.20641521	225.8907318	114.7721627
Lidoflazine	121.0510498	115.5360186	0	159.718684
Tropisetron HCl	128.1198809	116.8981882	213.4206292	131.4433305
Betaxolol hydrochloride	84.28123171	121.4377244	237.1230132	170.7948114
Cefixime	113.8742412	118.1098656	236.206663	#VALUE!
Nicardipine hydrochloride	107.9105779	110.7603756	125.4023358	134.7614333
Metrizamide	98.8373799	110.2820417	253.4028739	152.2624807
Probucol	77.19870111	101.5359065	163.8497874	87.73007585
Quetiapine	105.5726624	87.17645694	247.7209824	138.0869346
Mitoxantrone dihydrochloride	110.4459975	81.19147072	63.96029337	31.30712843
Tosufloxacin hydrochloride	52.99350485	53.69500755	0	39.98029373
GBR 12909 dihydrochloride	25.94266287	84.62775282	0	0
Efavirenz	22.84793928	19.96203285	0	0
Carbetapentane citrate	81.6316063	37.89778604	191.6737129	95.80883134
Rifapentine	0	0	0	0

Dequalinium dichloride	64.76570104	0	112.3478712	70.59667182
Neostigmine bromide	83.82446671	52.65684294	234.1791569	113.8808272
Ketoconazole	108.5202675	54.87356717	0	0
Niridazole	65.88770724	36.30059816	0	49.87082586
Fusidic acid sodium salt	0	0	0	0
Ceforanide	84.63957288	23.79566619	0	103.4236201
Ciclopirox ethanolamine	91.15667349	112.8948781	0	0
Mizolastine	99.11294521	101.2273021	0	0
Probenecid	130.624852	116.9270544	254.4583712	258.9011114
Amisulpride	110.0261928	88.32402291	256.2029238	127.1472674
Betahistine mesylate	95.62848734	115.3640702	244.8073052	136.6535209
Pyridoxine hydrochloride	112.213721	118.650213	242.0043399	147.9177248
Tobramycin	101.6782402	84.41802219	210.2772713	114.0418225
Mercaptopurine	93.28081065	116.7242275	313.0949646	136.9468772
Tetramisole hydrochloride	106.7838862	116.7966564	222.817687	138.1988617
Cytarabine	123.7220977	104.9581056	233.7632647	133.1599462
Pregnenolone	77.32282569	107.2948061	262.885055	95.78384952
Racecadotril	83.62688917	46.34060584	252.1259293	103.0247842
Molsidomine	82.24475272	32.10906327	166.0988882	180.7835973
Folic acid	98.14422866	48.33484264	238.3205344	132.6181379
Chloroquine diphosphate	128.2730315	50.27031692	195.7393507	137.1735758
Benazepril HCl	107.8015951	30.79112636	181.2257113	106.5431314
Trimetazidine dihydrochloride	72.56993652	36.55110817	201.5698512	104.2258835
Aniracetam	96.89927266	38.78312197	197.035721	91.9812598
Parthenolide	19.59164026	34.51897003	245.3128651	39.74834857
Dimethisoquin hydrochloride	93.97049939	45.59893312	107.473999	74.94842525
Terbutaline hemisulfate	136.4200432	108.5259596	250.9573105	160.6735214
Vatalanib	127.9651076	99.87653566	149.9647517	145.5283702
Ketanserin tartrate hydrate	120.9028991	108.5197021	226.6396565	145.4525303
Itopride	144.7949433	107.4628965	232.1826672	230.9997372
Hemicholinium bromide	135.6884552	94.23414374	226.2554055	145.7051743
Cefotetan	166.5474545	84.37386609	0	0
Kanamycin A sulfate	131.5789884	112.4280428	210.7627227	111.447448
Fentiazac	74.62269956	61.67237579	0	47.67309575

Amikacin hydrate	136.0003994	90.96002998	99.04893598	53.89892899
Brompheniramine maleate	124.0734655	64.58668367	121.5720285	66.6794439
Etoposide	121.4657642	97.78730961	0	124.4007043
Primaquine diphosphate	107.469775	81.82508915	0	26.92061245
Clomiphene citrate (Z,E)	9.418286971	6.47299338	0	0
Progesterone	153.8599488	67.85081804	214.9247607	101.0379643
Oxantel pamoate	116.2897211	38.17168367	243.929392	51.26279323
Felodipine	9.802412558	14.20021495	0	6.474024491
Prochlorperazine dimaleate	78.98917857	46.44061162	0	0
Raclopride	161.6662478	44.29073754	358.792376	109.7651236
Hesperidin	138.5483394	42.87890726	246.4339126	125.5485999
Closantel	126.8798789	0	0	88.72251111
Hexetidine	107.5787402	100.4446932	0	104.2681207
Alendronate sodium	134.1295969	102.8664463	183.506932	138.7545995
Selegiline hydrochloride	138.796604	111.5690707	178.3057239	225.6101358
Dipivefrin hydrochloride	135.6318822	86.65744367	188.9825374	93.55908116
Pentamidine isethionate	136.1514582	99.5441254	174.1887925	129.2860939
Thiorphan	154.9950631	100.9771899	210.7369407	113.4624512
Tolazamide	137.788707	75.07613518	161.3710408	123.9507294
Tomoxetine hydrochloride	118.9498078	99.88760221	63.01503702	122.58012
Nifuroxazide	113.6326288	94.47244615	0	47.69142486
Aceclidine Hydrochloride	135.4775814	102.3604896	199.8935548	91.38740326
Mirtazapine	108.9477944	103.5329748	178.0424684	111.6679438
Penciclovir	140.9987916	89.51389138	196.0850259	110.7600465
Dirithromycin	118.2427789	42.42432134	178.7568832	91.72755075
Levetiracetam	137.1977278	73.36209661	195.1720557	131.98619
Gliclazide	143.3526059	104.9806284	176.5248562	120.624068
Dexfenfluramine hydrochloride	138.9322136	36.06066278	223.8193956	94.03707363
DO 897/99	81.38709335	40.78043973	217.3474459	93.67123818
Etoricoxib	145.4198211	43.21596142	243.0743859	99.38007182
Prenylamine lactate	32.5249579	31.71897909	0	0
Sertindole	41.54790063	10.18465191	0	0
Sulmazole	70.21838077	80.14945901	146.6801739	90.33383127
Althiazide	54.99334852	106.0109384	172.475782	96.69575935

Gefitinib	71.61455417	76.86093157	55.52043161	60.17160121
Isopyrin hydrochloride	74.86055636	104.1332758	171.4203665	97.74802472
Flunisolide	75.28508951	95.56240792	176.3728714	139.1786916
Phenethicillin potassium salt	0	65.03439927	0	0
N-Acetyl-DL-homocysteine Thio	63.44840986	70.54213266	199.2551463	124.7886787
Sulfamethoxypyridazine	78.38979684	95.30602331	172.5907274	102.2094015
Flurandrenolide	65.68172142	68.15090909	222.3093452	125.8813267
Deferoxamine mesylate	69.47966701	57.53316128	0	113.3443038
Oxiconazole Nitrate	0	0	0	0
Mephentermine hemisulfate	68.42578926	81.9837677	161.3014508	84.60388301
Rebamipide	64.50074387	81.6911127	156.1109413	93.53983436
Liranaftate	65.55552563	106.8240218	133.2486005	62.14225721
Nilvadipine	62.77067729	112.1063758	41.37882073	50.17468498
Sulfadimethoxine	76.34249268	135.680957	145.4518926	79.78574852
Etanidazole	88.25161517	139.2592775	159.1273477	48.34442507
Sulfanilamide	89.12294035	100.5112151	148.0982438	91.16550589
Butirosin disulfate salt	17.10385757	85.60032591	0	0
Balsalazide Sodium	56.0652873	104.7472763	144.5300483	103.9033407
Carbinoxamine maleate salt	62.82369549	71.88231752	230.4341421	105.1579069
Niacin	83.79723359	68.65363836	250.6242604	109.7463774
Methazolamide	94.95368993	97.78253185	212.5566113	116.4311099
Bemegride	82.7497683	63.2672604	297.078749	127.4473234
Pyrithyldione	98.52878782	90.66155737	200.3265111	110.3076086
Digoxigenin	76.07172486	69.93636133	219.5200007	120.4219171
Spectinomycin dihydrochloride	67.24268649	117.8719307	115.0454708	83.60208153
Meglumine	93.80944748	55.8292262	251.0738231	86.09269099
Piromidic acid	94.61202057	85.2519013	180.2015197	90.86017321
Dolasetron mesilate	79.22181391	78.44148909	130.1560448	95.28031131
Trimipramine maleate salt	53.89378523	84.08496335	85.22656339	76.17992934
Clioquinol	0	44.09955258	0	0
Chloropyramine hydrochloride	68.09930614	68.72156514	137.2075066	67.40398385
Oxybenzone	82.63048988	80.22787913	148.0985477	73.12283797
Furazolidone	64.2315365	119.4713175	0	52.6700015
Promethazine hydrochloride	104.5553615	82.74211833	140.6685658	121.6081676

Dichlorphenamide	76.10842696	87.95930534	156.6501655	104.4480262
Diacerein	65.38310581	64.44410064	0	56.47633389
Sulconazole nitrate	0	0	0	0
Esmolol hydrochloride	74.08514045	82.76726379	168.7623978	167.9431226
Glimepiride	87.08300078	102.190293	108.185951	110.9622822
Sulfaquinoxaline sodium salt	72.29435359	109.0202935	189.9915513	66.59316446
Picrotoxinin	85.63733978	101.5035803	210.9258874	108.5894212
Streptozotocin	82.46673924	0	0	0
Mepenzolate bromide	99.08009858	106.7245962	156.4178235	116.4217702
Metoprolol-(+,-) (+)-tartrate salt	90.73643006	47.04254945	170.7867615	148.1103307
Benfotiamine	102.5377548	88.0418347	219.1466449	141.9345559
Flumethasone	89.95013606	106.1675491	145.2930916	154.7665178
Halcinonide	95.05994031	107.7601354	174.0664096	113.3900267
Flecainide acetate	77.12690962	64.30577004	164.4715535	110.650692
Lanatoside C	83.90877656	137.8602806	150.8916138	97.75690144
Cefazolin sodium salt	86.43280884	93.1446732	235.0378398	110.6029199
Benzamil hydrochloride	56.94773409	94.49214031	222.6290603	118.3592852
Atractyloside potassium salt	96.62420143	86.72305975	233.3460942	82.21609822
Suxibuzone	56.7339055	87.35695998	164.3331555	61.09179202
Folinic acid calcium salt	83.61584106	102.8924324	231.2451844	103.6286617
6-Furfurylaminopurine	99.13511944	145.5303973	124.9122852	89.39058838
Levonordefrin	92.32414515	103.3393017	132.7057679	63.02670659
Avermectin B1a	100.1719339	97.20096883	83.62662336	57.12625152
Ebselen	73.40568149	61.49435532	186.4627747	88.48116138
Auranofin	7.616406913	0	0	0
Cortisol acetate	97.84179366	72.95825657	304.5694716	113.2348124
Cromolyn disodium salt	104.3567626	85.24454615	175.6774754	114.0704637
Flubendazol	97.18125814	56.65819777	199.5081229	96.89900201
Bucladesine sodium salt	91.66507818	85.72170189	124.9306938	64.46703831
Felbinac	76.28799078	75.1781154	133.0393352	95.46235614
Cefsulodin sodium salt	95.19524573	110.1096022	112.5178621	153.8073111
Butylparaben	96.26081438	58.55794734	128.2643258	65.94065421
Fosfosal	113.6470795	113.5101883	145.4457388	106.9734187
Aminohippuric acid	71.87684492	79.55067925	222.2807723	122.7750024

Suprofen	60.0529123	105.8386978	123.3524666	95.60735369
N-Acetyl-L-leucine	86.35759195	58.61060999	199.6241973	70.65509331
Deflazacort	75.50112144	67.38891542	286.7758605	99.63166807
Pipemidic acid	85.54678779	101.4289964	204.5193944	93.08843368
Nadolol	75.78378933	117.358474	212.1864059	101.4506931
Dioxybenzone	72.17440474	71.97772983	125.8742603	90.52963789
Moxalactam disodium salt	97.94508754	110.6979129	0	0
Adrenosterone	98.19232665	83.91834359	225.2330948	84.84551111
Aminophylline	92.93106994	64.96441257	234.2447774	83.50021113
Methylatropine nitrate	88.97482997	47.72257214	208.9235544	150.6078989
Pranlukast	119.6674096	111.1452282	256.0050929	110.7342579
Nadide	78.32973742	138.3178826	219.3872412	140.5469707
Penicillamine	75.01012136	99.75688572	241.4251944	92.41175678
Sulfamethizole	100.175237	129.2064996	211.0181745	105.0999328
Zileuton	80.5906441	98.98784492	130.0353759	72.63708509
Medrysone	97.62391021	83.68194467	234.7746425	118.6162518
Loratadine	104.1551008	72.60933591	0	55.04741946
Flunixin meglumine	70.49923868	129.673017	15.07781108	16.89278701
Tetraethylenepentamine pentah	121.867538	116.3735643	167.9794823	97.83969218
Spiramycin	154.1852384	94.54127226	0	0
Nisoldipine	68.0524688	113.2568545	0	0
Glycopyrrolate	122.4836145	86.90765462	213.5975415	79.58973211
Acefylline	95.08731836	101.2375408	216.6501313	99.05795394
Aprepitant	41.65582534	63.38660062	0	0
Acitretin	52.25098403	61.40623409	0	0
Monensin sodium salt	17.6400254	46.7727975	0	0
Zonisamide	112.1985706	111.2338285	192.5232786	117.0333883
Isoetharine mesylate salt	121.0298491	89.33677051	189.8239781	88.12099053
Irsogladine maleate	118.5304721	95.11114365	203.1960166	80.23026479
Mevalonic-D, L acid lactone	0	0	0	0
Azlocillin sodium salt	0	76.65069541	0	0
Hymecromone	80.89676859	85.44794356	266.1889762	92.18649229
Clidinium bromide	97.01119983	89.52669096	251.351845	103.8179943
Abacavir Sulfate	85.18716299	68.01625838	278.7897483	106.4951147

Sulfamonomethoxine	109.0525775	101.0981256	203.7838108	108.3307066
Diloxanide furoate	67.34171516	61.38717662	105.2976246	56.5362209
Benzthiazide	102.1899722	104.1237383	187.9629947	103.424536
Metyrapone	116.0075019	56.80926339	187.5555785	69.93285254
Trichlormethiazide	127.8847176	128.1387217	112.7766981	109.0707742
Urapidil hydrochloride	109.1291594	90.50367515	293.4341595	117.8945738
Oxalamine citrate salt	96.88140856	96.98587221	156.2069435	145.0736813
Fluspirilen	81.26861376	59.53002569	0	21.58945572
Propantheline bromide	95.00638337	56.55909859	183.7476477	84.9968024
S-(+)-ibuprofen	94.81446735	81.06368286	228.1867792	68.89795364
Viloxazine hydrochloride	92.64095374	103.0988844	232.9754795	126.8558036
Ethynodiol diacetate	127.6040269	66.11429963	262.4979363	84.7565594
Dimethadione	98.60904383	102.7670823	229.1291907	114.7875061
Nabumetone	72.15519847	89.93975851	98.41981787	68.79829279
Ethaverine hydrochloride	93.97821973	67.74395856	217.9692704	95.91463881
Nisoxetine hydrochloride	112.3000611	64.85999263	233.364156	102.1818224
Dydrogesterone	111.410651	150.2617184	305.2703511	85.24400728
Terazosin hydrochloride	104.3611677	161.7981905	227.4427501	118.6161394
Sumatriptan succinate	93.45688466	153.0086867	268.7439476	97.84825373
Phenazopyridine hydrochloride	106.1021397	151.10755	139.4286947	89.25433129
Opipramol dihydrochloride	104.676502	152.8698162	164.4208988	89.93414517
Demeclocycline hydrochloride	0	0	0	0
Nalidixic acid sodium salt	83.58022916	64.89961557	156.8931014	102.9915784
Fenoprofen calcium salt dihydra	89.25547287	127.360226	161.1332705	108.3012355
Oxacillin sodium	88.18713598	121.4004163	0	186.9734927
Piperacillin sodium salt	43.71776783	57.52286768	0	0
Beta-Escin	111.4934213	116.0347422	140.6787598	157.5525999
Diethylstilbestrol	119.5805629	75.12637949	0	101.352528
Thiamine hydrochloride	106.0543826	77.27853298	166.1383693	88.24990976
Chlorotrianisene	109.8684099	60.01216167	166.1113287	91.84936607
Tazobactam	90.9185277	65.30584926	174.27769	231.0244271
Ribostamycin sulfate salt	116.6085838	77.29397209	213.6951288	135.8200988
Ibandronate sodium	124.7322347	117.7676405	170.5629933	109.5650166
Methacholine chloride	95.33737215	76.35929434	113.6161412	98.72259711

Warfarin	78.97152871	64.14136166	74.46153322	54.61304034
Pipenzolate bromide	79.29162884	58.35292293	174.6897831	114.4812404
Butacaine	103.0038243	156.8058888	279.816131	111.7675188
(+)-Isoproterenol (+)-bitartrate s	100.8982951	86.7875037	257.7218388	110.4132294
Cefoxitin sodium salt	107.3309068	149.9220771	172.5774398	102.1384902
Monobenzone	106.8887116	75.0750413	410.3663802	84.6855508
Ifosfamide	93.92928404	130.5211566	248.9222395	110.9720569
2-Aminobenzenesulfonamide	83.46735226	114.0258345	265.971156	0
Novobiocin sodium salt	14.85656682	48.85892025	0	0
Estrone	95.44095199	76.3038281	292.4157758	125.438426
Tetrahydroxy-1,4-quinone monc	141.8434953	129.6989592	237.5747583	186.7026879
Lorglumide sodium salt	64.73496436	112.3359244	27.65587252	30.65583849
Indoprofen	83.53149647	86.20431972	147.3952015	125.5978415
Nitrendipine	92.29299364	52.43878143	28.08574859	31.06716729
Carbenoxolone disodium salt	31.40617169	66.80190658	14.30713642	0
Flurbiprofen	90.88193852	95.92162453	131.5410144	65.62527526
Iocetamic acid	91.7859282	121.8916463	336.7222636	191.3465301
Nimodipine	121.8373307	49.69098643	49.4832511	90.76312336
Ganciclovir	113.4145312	116.0120384	168.0314976	190.6147857
Bacitracin	43.55813078	70.23193933	51.09630601	0
Ethopropazine hydrochloride	113.0222521	45.45355739	94.06239878	59.68191012
L(-)-vesamicol hydrochloride	49.88676204	38.28605493	0	10.43878839
Pranoprofen	130.4571793	146.9717782	234.599553	73.33021535
Butamben	122.7989899	178.1363575	212.0370849	96.68105675
Secnidazole	47.08197448	29.15444812	0	104.8678781
Sulfapyridine	141.0858623	152.1720528	229.9845256	99.49788577
Pempidine tartrate	137.1231437	119.366243	201.7451242	103.0953664
Meclofenoxate hydrochloride	126.5732664	56.02049298	208.6232202	118.8193126
Clodronate	145.7227759	54.66443927	230.0072811	156.1417397
Furaltadone hydrochloride	146.7477876	140.7221826	0	95.32928444
Ibutilide fumarate	141.4973039	91.9173966	166.2163544	156.7417693
Ethoxyquin	137.7093435	60.54481568	141.0487622	84.13263994
Thimerosal	135.4901528	94.23694168	0	113.744588
Tinidazole	65.98157367	52.71884501	0	43.08828717

Tramadol hydrochloride	137.8463128	77.75973481	204.0762497	152.881642
Guanadrel sulfate	150.2588469	55.54611228	243.6283027	128.8408667
Estropipate	142.582894	101.6994116	209.2548333	178.161762
Vidarabine	142.4503265	98.41642806	231.7274406	182.679191
Butylscopolammonium (n-) bror	145.1484398	91.05721197	210.8409591	97.87442388
Sulfameter	148.1049135	80.89826197	172.7985446	181.841994
Irinotecan hydrochloride trihydra	145.490125	79.46354974	229.2829638	154.0656221
Isopropamide iodide	108.6501423	62.52665235	196.6253286	80.92469572
Olanzapine	124.3582831	146.7961338	230.7729326	124.1700941
Nizatidine	123.748691	116.8005488	220.5124188	116.3170417
Trimeprazine tartrate	123.2674005	125.577861	95.07499349	86.62182679
Thioperamide maleate	123.1806569	111.592805	307.7635985	117.0016162
Nafcillin sodium salt monohydra	172.7186935	85.86979079	0	0
Xamoterol hemifumarate	128.1492608	94.21481053	213.4407541	109.098074
Procyclidine hydrochloride	142.9519489	123.3293045	130.731193	112.7202413
Rolipram	143.1849358	81.01752354	193.3321004	124.9986443
Amiprilose hydrochloride	154.0135176	99.66999015	175.3381826	202.5927865
Thonzonium bromide	14.23007623	0	0	0
Ethynylestradiol 3-methyl ether	135.729562	75.97623986	206.1882901	164.028957
Idazoxan hydrochloride	127.3304374	55.66466189	224.1165343	0
(-) -Levobunolol hydrochloride	134.8537014	57.09328723	247.8130878	140.5843846
Quinapril HCl	141.0510569	117.5401733	250.4169816	133.8501356
Iodixanol	124.1844034	123.6939261	258.1070071	224.6457673
Nilutamide	139.7851686	44.93878396	213.4914618	153.7037483
Clinafloxacin	134.2773834	94.22098714	0	181.4539433
Ketorolac tromethamine	137.0671198	66.70701435	272.4242555	193.7822384
Equilin	130.5926106	51.13822488	380.2664792	117.6074255
Protriptyline hydrochloride	114.2449186	45.84514216	103.1933155	106.2230086
Tylosin	0	0	0	0
Alclometasone dipropionate	103.6054841	152.4085597	321.0571225	112.2641078
Citalopram Hydrobromide	114.5290819	142.9768676	171.7230904	122.3440621
Leflunomide	83.30889839	111.9793329	22.72970033	21.80229152
Promazine hydrochloride	106.9315248	133.7611664	102.1986264	121.4622899
Norgestrel-(-)-D	119.8821485	65.05095719	239.1913225	98.20038316

Sulfamerazine	135.0707737	80.00095251	223.7706685	192.091224
Fluocinonide	144.410068	117.0173311	234.6368122	147.1262376
Venlafaxine	112.7909658	116.0520558	194.406179	185.2927258
Sulfamethazine sodium salt	111.0505073	84.03334677	172.8103203	203.2892199
Ethotoin	112.5109898	113.6980613	221.7741288	187.9547966
Guaifenesin	121.7002619	119.4091694	246.73147	188.9191084
3-alpha-Hydroxy-5-beta-andros	124.2770023	87.08532806	274.8170695	159.3387432
Alexidine dihydrochloride	0	0	0	12.48648549
Tetrahydrozoline hydrochloride	126.8225917	113.3579356	212.8302633	179.4048481
Proadifen hydrochloride	108.9082359	141.1830861	90.19871467	119.4967661
Hexestrol	25.21146359	31.47519302	0	0
Zomepirac sodium salt	135.220727	98.06664861	187.3118976	182.4086208
Cefmetazole sodium salt	139.8666441	83.7853457	396.8375972	201.3214251
Cinoxacin	87.18136387	91.84345712	206.7823727	106.3428509
Paroxetine Hydrochloride	80.25891479	53.19964831	87.92710569	0
Propofol	110.0265509	109.6029034	149.2189432	91.44051377
Nylidrin	151.4345577	115.7695506	224.4594253	117.7426803
S(-)Eticlopride hydrochloride	140.3366505	98.0105576	235.3719551	114.9348568
Liothyronine	54.61790038	103.1519241	53.40690153	15.4792112
Primidone	118.7240389	103.0467456	215.9209063	123.0903478
Roxithromycin	124.6110417	63.53474848	185.6455383	98.08861402
Flucytosine	115.607399	89.24864258	195.9771411	153.8186869
Beclomethasone dipropionate	171.3405417	146.8957926	248.8075674	215.4131922
(-)-MK 801 hydrogen maleate	117.9104068	112.5698478	378.6832147	180.1771429
Tolmetin sodium salt dihydrate	110.1635184	124.5237241	199.5443716	184.1057031
Bephenium hydroxynaphthoate	101.8453649	72.06894035	122.4321175	128.4614215
(+) -Levobunolol hydrochloride	108.2626585	73.13336546	265.478042	142.075103
Dehydroisoandosterone 3-aceta	134.053073	115.6040319	218.7526154	99.77642512
Doxazosin mesylate	108.0371438	137.4669173	78.13743512	0
Benserazide hydrochloride	129.0144561	60.2956646	254.333319	162.8931901
Fluvastatin sodium salt	68.19606317	114.1791704	24.77254486	0
Iodipamide	118.711172	94.26624508	183.5941965	208.7065538
Methylhydantoin-5-(L)	135.4595198	58.7799627	259.6238606	157.3557843
Allopurinol	118.1719065	53.10143719	230.5131592	139.9626287

Trihexyphenidyl-D,L Hydrochlor	94.80045935	104.5406006	99.32135036	68.36036885
Clobetasol propionate	194.6600313	141.5614673	389.9668346	145.2483811
Succinylsulfathiazole	116.7962175	134.4423378	231.3112492	91.86918248
Podophyllotoxin	110.9042053	131.497709	211.881264	91.50098252
Famprofazone	129.1837039	113.5907813	164.9080423	106.6270438
Clofibric acid	113.4701063	78.86227387	243.75837	94.60509325
Bromopride	105.2633148	84.27621227	250.3046227	167.5235173
Bendroflumethiazide	125.6544086	140.4959103	107.103412	160.14131
Methyl benzethonium chloride	0	0	0	0
Dicumarol	63.07620612	96.63660149	19.9650621	41.3832985
Chlorcyclizine hydrochloride	116.4251113	82.55720216	72.54898465	122.8560433
Methimazole	136.9541212	116.4552169	225.5085437	154.0099907
Diphenylpyraline hydrochloride	128.8288767	113.4869623	151.353399	124.8699193
Merbromin	10.94591033	19.44822319	0	0
Benzethonium chloride	0	0	0	0
Hexylcaine hydrochloride	122.6649553	93.61094835	181.3943853	119.417156
Trioxsalen	126.4953878	111.5581434	213.4249755	97.07253821
Drofenine hydrochloride	120.9812314	104.0169587	120.0577351	126.0806265
Doxofylline	111.5422992	79.3579675	212.7745208	145.2243528
Cycloheximide	108.5313198	94.44966951	240.1027982	100.031795
Gabapentin	108.8535162	102.5565431	234.7371667	104.0663666
Pentetic acid	120.8627732	98.39665721	134.8571466	85.85183594
Raloxifene hydrochloride	70.4369666	110.0462961	0	0
Bretylium tosylate	122.340862	82.7634183	267.0228753	105.6212795
Etidronic acid, disodium salt	101.7003853	103.8888627	190.6418791	100.7038339
Pralidoxime chloride	101.9547125	95.45852173	230.417838	125.8235487
Methylhydantoin-5-(D)	116.3213281	103.3099213	252.7888358	116.3225558
Phenoxybenzamine hydrochlori	136.9956919	116.1474133	165.6983216	134.6609286
Simvastatin	108.6771504	112.259675	0	0
Salmeterol	120.5763161	107.8041209	116.5880484	114.3779779
Azacytidine-5	123.8593622	97.3349055	232.9225045	184.2989506
Altretamine	141.2000437	65.01814961	255.81844	194.6351586
Paromomycin sulfate	114.5189655	73.4574409	228.3880861	148.3790954
Prazosin hydrochloride	139.0281585	99.21697577	174.9879542	122.2288846

Acetaminophen	120.5713537	115.7485321	239.9535592	163.043774
Timolol maleate salt	126.6438253	72.4486082	263.9354681	138.0451534
Phthalylsulfathiazole	119.4097883	111.3550544	222.8813889	213.1126125
(+,-)-Octopamine hydrochloride	136.6837994	101.1874607	214.2836923	161.1820486
Luteolin	38.84516075	42.7228322	0	9.549107185
Stavudine	108.7118608	52.45262522	221.7993095	110.6114351
Sulfabenzamide	118.1632876	102.8645877	157.3160882	73.42198705
(R) -Naproxen sodium salt	129.7638764	134.6373654	195.1598835	115.464156
Benzocaine	128.3893748	114.5489097	226.8167238	130.3566032
Propidium iodide	104.4735131	107.850671	93.50359847	28.02651764
Dipyrone	140.6178974	108.6274563	179.3061313	162.2047725
Cloperastine hydrochloride	101.3438295	47.5668861	85.04499764	61.81402428
Isosorbide dinitrate	121.3942443	84.37505076	67.22794079	101.540557
Eucatropine hydrochloride	153.577817	147.2075683	189.4571549	166.9801516
Sulfachloropyridazine	140.4058995	110.0712307	208.337552	141.563874
Isocarboxazid	128.5875518	90.43615008	207.9271291	146.8809648
Pramoxine hydrochloride	151.1281123	128.5587033	238.7859612	137.5076962
Lithocholic acid	147.6907439	111.3977833	34.65157906	150.9164257
Finasteride	146.1536657	92.86219075	222.268409	180.8618329
Methotrimeprazine maleat salt	126.8915476	74.44571752	107.7982159	121.1307409
Fluorometholone	138.6244752	91.72149823	238.3961761	179.5772627
Dienestrol	36.83257742	88.51606061	0	0
Cephalothin sodium salt	139.3219207	102.2445057	249.6466834	0
Pridinol methanesulfonate salt	150.8110915	87.52889307	169.307837	140.3372237
Cefuroxime sodium salt	139.5703163	61.19132928	269.9926663	155.2802247
Amrinone	117.796217	106.4386259	240.6244063	95.13315858
Iopamidol	126.7383004	116.2305706	173.3937586	117.5183915
Crotamiton	131.6430805	105.1897289	132.0348505	110.8651832
Iopromide	141.0054326	106.8485721	175.9459263	112.2829732
Toremifene	138.5532608	96.99201894	0	112.0090077
Theophylline monohydrate	128.7680404	102.8838583	198.6416897	138.9317863
(R)-(+)-Atenolol	125.1228081	117.229145	173.514745	130.7899134
Theobromine	142.2154213	63.47932982	180.4823669	130.7529518
Tyloxapol	139.4635681	107.142291	132.8999717	168.3736483

Reserpine	137.5300055	119.3771728	151.3850578	155.2821787
Florfenicol	0	0	0	0
Bicalutamide	144.7722211	104.1565592	170.5919629	123.3388209
Megestrol acetate	139.3580689	81.58148115	127.1283859	158.0820906
Scopolamine hydrochloride	122.0147656	70.85678758	188.5715888	158.2329649
Deoxycorticosterone	161.1819917	117.4479303	277.083707	131.0293876
Ioversol	131.9608453	121.7292122	197.6684701	178.1728707
Urosiol	178.6349731	69.31710533	259.2562703	155.2179422
Rabeprazole Sodium salt	133.442466	119.6160697	181.0293836	135.1238004
Proparacaine hydrochloride	144.6270773	99.6092652	214.1139245	136.3575429
Carbachol	143.1908395	66.08509859	208.0144414	167.5610881
Aminocaproic acid	124.7158902	62.72119806	190.3817516	117.1722923
Denatonium benzoate	78.26202531	63.76286297	141.3094641	103.2051809
Etomidate	75.50944888	67.88563792	226.2479725	120.1365182
Canrenone	71.18040867	68.74953811	186.6088248	67.44641767
Tridihexethyl chloride	73.33962148	64.04948105	153.6419938	78.08756917
Enilconazole	79.70140321	73.84330321	0	0
Penbutolol sulfate	39.75368859	52.26091454	155.914386	0
Methacycline hydrochloride	0	0	0	0
Prednicarbate	67.43421879	64.6264252	179.4211969	25.38357135
Floxuridine	67.93313317	72.89592488	99.71674922	91.44751843
Sertaconazole nitrate	0	0	0	0
Sotalol hydrochloride	74.27573624	80.87392683	180.4166009	108.6793206
Repaglinide	36.01086582	64.85170493	44.39434546	0
Gestrinone	39.27803274	59.07003801	246.5534602	14.10956201
Piretanide	66.3682545	70.74730116	151.3670618	98.12134473
Decamethonium bromide	66.4217709	71.68479573	194.3419164	111.6406112
Piperacetazine	81.4720859	83.95867898	106.7948009	76.34789026
Darifenacin hydrobromide	95.53230756	66.30962002	83.92563396	88.98110926
Oxyphenbutazone	0	32.70843295	19.42386162	0
Roxarsone	40.71664631	57.46418295	23.6013936	0
Quinethazone	63.06122708	68.16629478	145.921809	92.42332506
Buspirone hydrochloride	79.54781248	86.96267533	204.8008634	98.24242365
Anastrozole	58.00009459	52.30461473	191.1481731	81.41500621

Doxycycline hydrochloride	0	0	0	0
Sulbactam	26.15038011	38.87765652	137.4284568	0
Fleroxacin	74.21721819	68.26735888	78.30883359	104.294082
Clavulanate potassium salt	72.85424363	66.2951362	204.2198077	110.1265781
Valproic acid	71.56707689	61.00645914	207.4862926	98.65679571
Mepivacaine hydrochloride	84.98537266	59.52368455	220.0545149	104.7973731
Rifaximin	0	0	0	0
Estradiol Valerate	58.21245901	62.03012364	93.07525211	0
Remoxipride Hydrochloride	70.62063378	75.61096967	114.6140718	110.5457924
Moricizine hydrochloride	79.3625742	78.25275854	83.16315011	79.24955507
THIP Hydrochloride	96.66590163	80.05798552	185.8620916	108.3204183
Iopanoic acid	74.0908875	64.803041	34.35310111	40.93342846
Pirlindole mesylate	58.23966114	59.60247102	57.89520369	61.84447211
Pivmecillinam hydrochloride	79.98476533	69.19848039	178.952197	96.12106772
Pronethalol hydrochloride	68.31092491	70.63086551	175.405445	107.9162145
Levopropoxyphene napsylate	56.92258033	56.21463321	196.448927	76.55185992
Naftopidil dihydrochloride	60.45058911	53.249665	112.8790785	0
Piperidolate hydrochloride	48.13459527	66.7636474	182.117864	103.84732
Tracazolate hydrochloride	63.09530373	64.60618448	115.3112264	66.22294252
Trifluridine	43.49402708	66.30650777	245.7777333	99.017944
Zardaverine	73.22979954	66.84543757	241.0764143	135.5502198
Oxprenolol hydrochloride	51.95376624	68.77813746	197.0797101	95.75072552
Memantine Hydrochloride	65.05793831	63.73209499	165.2549355	69.96444275
Ondansetron Hydrochloride	38.97263326	57.75388939	281.6811672	105.2750148
Ozagrel hydrochloride	50.07368454	81.21879148	193.1010689	90.56927719
Propoxycaine hydrochloride	54.5663072	60.04880872	266.4271749	87.71198332
Piribedil hydrochloride	111.1207925	55.8053373	192.1331934	141.8195762
Oxaprozin	48.13364552	55.553334	30.11070556	27.17597558
Acetylcysteine	82.83160062	104.2587852	150.3500455	102.3470791
Melengestrol acetate	46.80165918	69.46816766	71.15912197	38.77119252
Bromhexine hydrochloride	53.58698374	70.3550195	26.79835504	7.095931656
Anethole-trithione	33.73509479	57.61708509	65.87574459	0
Amcinonide	88.83944639	57.69490399	222.4593695	122.965182
Caffeine	65.26079219	68.73024004	223.2680242	93.28061465

Carvedilol	55.06607941	92.96631934	181.6524092	96.59734689
Methenamine	48.7655715	73.67966219	237.4322544	90.85695357
Phentermine hydrochloride	57.07304267	53.94053522	253.2410309	96.2946588
Diclazuril	9.775703679	45.76381402	0	0
Nitrocaramiphen hydrochloride	101.0251398	102.336852	129.5424323	100.7943571
Phensuximide	94.05809121	88.11979837	146.4036962	133.1076238
Nandrolone	84.23434046	85.33005387	201.2077146	89.28173102
Ioxaglic acid	80.15782964	77.53129729	137.5529585	88.21422007
Dimaprit dihydrochloride	92.09324339	75.81265212	153.1379097	106.8154482
Naftifine hydrochloride	89.39212159	75.51764305	168.3459341	38.70908099
Oxfendazol	99.95385302	77.86238705	212.4260183	113.1275559
Meprylcaine hydrochloride	53.22952552	60.49418453	194.0430785	98.47191282
Guaiacol	59.80714435	56.96687163	218.37162	117.5549563
Milrinone	42.16125224	62.18448841	203.8556671	134.0742445
Proscillaridin A	66.60213575	70.72659825	196.7089171	52.87184337
Methantheline bromide	45.26756271	64.90253911	229.4668978	82.68140826
Pramipexole	67.7786222	57.41371984	239.7620692	112.3125531
Ticarcillin sodium	50.81922255	49.85616024	0	0
Norgestimate	0	25.93815312	0	0
Thiethylperazine dimalate	21.35041538	53.64459669	0	0
Chlormadinone acetate	64.91066129	71.65348401	131.6780036	102.9574473
Mesalamine	56.78198178	60.95084399	248.2742525	86.21865192
Phenylbutazone	82.56945921	50.12105633	49.95859672	20.21349182
Vorinostat	55.84659779	66.52046198	152.7113146	124.9991692
Famciclovir	97.39925255	171.9364996	159.7377416	112.7449246
Dopamine hydrochloride	70.08232724	79.10038956	224.3953584	101.2720416
Cefdinir	110.6415134	60.88863758	249.9364873	156.9257863
Carprofen	54.76336594	60.64066072	18.87019644	25.83912276
Celecoxib	45.40245925	66.87579504	0	0
Candesartan	60.02331744	79.56703745	127.370718	41.40311496
Fludarabine	42.71360465	84.36332443	270.0041665	91.81549119
Cladribine	60.63829535	78.31742903	232.2237516	125.6758182
Vardenafil	61.65136967	56.77898699	250.9717494	107.3060921
Fluconazole	49.10451397	67.02981236	215.1835391	126.4205859

Gliquidone	57.80321038	107.6130348	25.81920081	34.83830975
Imidurea	85.93344444	118.153727	159.8269581	195.0607884
Pizotifen malate	86.19276464	81.12159308	87.64280173	70.00356914
Lansoprazole	82.40888043	76.69940466	76.38080585	75.29901526
Ribavirin	69.11237708	66.76407381	179.6118827	90.44870312
Bethanechol chloride	81.00079488	66.00421919	239.4594649	105.7405776
Cyclopenthiazide	57.01380421	86.56237753	141.3045163	64.38804033
Cyproterone acetate	55.44023138	74.57692499	148.7605011	115.9652927
Fluvoxamine maleate	58.05720544	66.14178566	218.6197128	79.83000504
(R)-Propranolol hydrochloride	40.38889157	69.83405695	250.4593717	104.1925383
Prothionamide	56.53359286	98.52562267	225.6179022	86.38446881
Ciprofibrate	44.9464572	66.24487254	237.7791296	70.68267072
Fluticasone propionate	55.41612262	73.38386911	289.5617899	159.3457447
Formestane	40.0713608	74.65206387	288.7352452	103.8371199
Zuclopenthixol hydrochloride	48.3793602	79.80043528	0	0
Benzylpenicillin sodium	44.22843655	48.28096545	0	0
Proguanil hydrochloride	57.69744539	83.96730376	135.9206437	101.381555
Chlorambucil	68.17185826	65.13777368	244.5196144	114.3722181
Lymecycline	82.63740104	64.93586577	0	0
Methiazole	68.11921574	70.38694604	297.0300362	120.2102149
5-fluorouracil	69.11096229	111.1219986	0	88.39539783
Mesna	84.88792059	118.1776807	252.8689548	115.7617028
Mitotane	39.91067403	60.005481	0	0
Ambrisentan	44.07643991	100.819494	171.8338557	76.60044499
Triclosan	0	0	0	0
Enoxacin	62.1138774	85.01929113	162.7179764	97.57018969
Olopatadine hydrochloride	46.39755865	114.137858	263.2529352	116.373009
Granisetron	72.8331458	113.5984013	285.8893317	96.02378566
Anthralin	71.5342247	67.24924534	25.8662544	109.3372668
Lamotrigine	68.07857822	61.53387672	233.2937739	79.33542583
Alfadolone acetate	112.6398727	133.2870923	269.190924	188.995082
(S)-propranolol hydrochloride	109.3528714	131.4451255	210.2055719	136.9405339
Alfaxalone	107.5390529	105.571246	292.4156625	85.68302796
(-)-Eseroline fumarate salt	98.03824481	101.1650722	202.1336767	49.72878088

Azapropazone	56.03412462	70.80855772	128.6610174	47.91106025
Isosorbide mononitrate	100.0553728	90.35711725	158.251915	64.8921701
Meptazinol hydrochloride	97.6283639	98.46915576	214.2691056	85.95976909
Levalbuterol hydrochloride	98.25722963	73.48103424	270.8979257	101.7801154
Apramycin	108.1112784	59.67655746	231.2016869	105.5694255
Topiramate	57.3231028	68.77933163	235.9177025	115.4844013
Epitiostanol	98.60485019	96.10819635	313.8545821	92.55952821
D-cycloserine	58.37001066	78.05669696	311.0305349	74.42533597
Fursultiamine Hydrochloride	78.68536484	90.5315062	206.3355765	106.3345491
2-Chloropyrazine	67.47360945	95.46170504	293.9180573	93.00725049
Gabexate mesilate	74.19415024	94.40390051	273.1410642	86.14198575
(+,-)-Synephrine	75.67402272	84.55494861	251.9065578	135.2320184
Pivampicillin	87.88355627	89.53100607	194.1267742	135.0844378
(S)-(-)-Cycloserine	88.31176053	65.7656968	479.6279766	144.0137189
Talampicillin hydrochloride	105.5870295	66.05361604	231.830684	94.4834187
Homosalate	103.6798477	92.07956741	139.0338876	84.04494687
Clofibrate	102.6200801	149.7074678	210.717557	103.7213124
Cyclophosphamide	98.82240812	110.2612767	240.971645	112.5325062
Aripiprazole	110.6320122	111.2190152	157.7288432	91.95932724
Ethinylestradiol	53.0301443	102.2916946	136.8574539	89.50592531
Fluocinolone acetonide	107.6303386	67.46307619	294.4082251	96.14434428
Sparfloxacin	82.85943495	113.2657	385.9814983	113.2247355
Desloratadine	73.77146507	98.83293221	134.4017502	60.39682344
Clarithromycin	60.45200216	67.86387606	188.9768778	85.85986985
Tripelennamine hydrochloride	76.57048261	78.20389157	284.7732624	101.4749291
Tulobuterol	107.9621509	80.85605251	256.1174479	93.94547923
Flucloxacillin sodium	48.9305612	104.9138013	0	0
Spaglumic acid	89.27388178	146.8702635	224.0295115	218.9813175
Trapidil	65.42398316	124.3438031	266.7484819	94.08112268
Ranolazine	74.3350152	94.23819588	215.3565568	94.41459087
Deptropine citrate	57.02806351	89.3741852	66.45803355	94.66361198
Misoprostol	89.34752225	89.17547758	221.3250575	118.6394115
Sertraline	48.73864378	111.8522837	0	0
Sulfadoxine	58.41597577	105.2759765	233.6604726	91.68833991

Ethamsylate	49.87287569	67.93886807	226.9579769	105.496933
Cyclopentolate hydrochloride	48.01788459	82.21672704	219.8327833	111.2730762
Moxonidine	46.44602255	110.9864739	268.6299106	106.2074399
Estriol	37.57178074	109.5544975	329.6025171	86.01337131
Etilefrine hydrochloride	35.05656869	105.6035075	267.2083484	115.5245801
(-)-Isoproterenol hydrochloride	61.68930602	128.3626644	272.4908444	103.3212574
Alprostadil	45.76750962	121.831552	174.0756533	29.00028633
Sarafloxacin	51.58201772	89.27528919	110.7769594	65.27878782
Tribenoside	37.18043136	102.541534	69.66897765	55.30789926
Nialamide	80.1774694	77.96711379	301.889176	152.1862859
Rimexolone	107.100313	69.56980747	236.8528917	104.6804212
Toltrazuril	52.27662782	103.6228697	128.4181773	43.30061112
Topotecan	87.54270497	108.1796974	206.0800891	105.6657214
Atorvastatin	61.56926957	127.0738293	58.40882472	44.01445519
Azithromycin	70.58725445	79.92433819	220.7269467	99.05139942
Ibudilast	44.77097406	127.421265	237.4798175	75.75232751
Losartan	70.97743901	74.91528521	140.6066496	66.5152948
Benztropine mesylate	47.2809233	102.6980773	170.0524747	120.7226578
Vecuronium bromide	56.03867458	118.6476353	336.0252351	81.86359165
Telmisartan	84.83056203	114.7078554	291.1228334	116.3672211
Nalmefene hydrochloride	79.04630836	114.6049236	285.3819787	139.8790976
Bifonazole	22.87483899	88.57123961	0	0
Isradipine	10.90411211	23.68340688	4.350931309	3.700770788
Perindopril	94.72820471	133.12874	272.5903318	138.5191566
Tiletamine hydrochloride	98.66281045	127.4443942	323.5337562	81.26058755
Fexofenadine HCl	100.4201942	97.85878798	277.0985701	85.4913834
Isometheptene mucate	74.64525148	107.632731	240.5136671	144.0166921
4-aminosalicylic acid	82.15007149	95.25528638	217.9184448	113.9665182
Nifurtimox	66.09776036	96.70324329	0	20.29834347
Clonixin Lysinate	54.30081507	78.302784	62.01665229	31.61280025
Letrozole	50.38736088	62.78492998	228.179456	91.74671931
Verteporfin	31.36830015	67.05313747	153.6999534	66.81312779
Arbutin	62.89945238	114.6388965	204.2212554	98.82772802
Meropenem	33.02343917	107.6414573	0	77.06950593

Tocainide hydrochloride	39.65044722	134.1773363	296.3033898	122.3421994
Ramipril	49.69786174	115.91249	324.4272497	97.93814856
Benzathine benzylpenicillin	25.26558333	97.44149909	0	0
Mephenytoin	49.72347253	102.5314289	254.9835444	109.9413805
Risperidone	57.13569978	115.0939845	508.2741539	163.9150611
Rifabutin	0	0	0	0
Torsemide	86.1312891	55.38634043	170.9640553	79.00985054
Parbendazole	52.75276607	115.5005205	212.5764293	95.80097427
Gatifloxacin	82.20005155	98.62309035	143.1425278	81.43535116
Bosentan	0	129.5948573	0	0
Gemcitabine	103.3132299	100.7948919	302.7925941	135.7983629
Olmesartan	30.88079043	107.0307477	85.8204002	24.90858599
Racepinephrine HCl	95.18550903	74.24129427	268.5104902	95.64906789
Montelukast	48.49918276	100.1485463	70.79016802	63.40492725
Docetaxel	50.03331924	134.6626921	177.3018613	80.82972983
Cilnidipine	69.79416818	109.0508349	115.6836755	143.8908071
Imiquimod	84.581815	113.2493016	323.9989857	89.71999423
Fosinopril	62.24899372	89.80820698	97.64846776	39.8355313
Halofantrine hydrochloride	12.57695331	11.4174219	0	5.310066762
Mecamylamine hydrochloride	114.6722374	138.1881518	300.6411578	146.622915
Articaine hydrochloride	110.5356563	133.6823191	281.5535874	106.1072177
Procarbazine hydrochloride	117.4148008	104.1769152	272.6706855	98.80551202
Nomegestrol acetate	87.19701253	91.54400585	216.6341296	114.1906161
Viomycin sulfate	104.6055434	112.0206731	261.7885443	131.1532708
Pancuronium bromide	94.34385629	104.0014427	286.5636435	122.3964593
Saquinavir mesylate	84.81006726	93.91598047	41.49944129	27.85227238
Molindone hydrochloride	92.34790042	82.01762581	282.5617174	90.25051759
Ronidazole	13.17684459	23.39633952	0	0
Alcuronium chloride	111.4271054	129.2657046	250.8047179	142.1505314
Dorzolamide hydrochloride	49.42804205	97.99510148	255.8076439	89.96165425
Zalcitabine	106.6508917	121.3440371	279.5642462	135.9904299
Azaperone	66.84485561	126.8739401	377.9130963	130.8596288
Methyldopate hydrochloride	106.3389159	116.7971684	303.0110266	123.2012007
Cefepime hydrochloride	87.41626692	115.372987	300.9751988	150.8544994

Levocabastine hydrochloride	107.1982584	132.9925535	267.3022321	176.0530848
Clocortolone pivalate	103.0847255	90.75355486	239.345838	163.5025904
Pyrvinium pamoate	73.48203247	67.54391134	55.31463601	23.34010378
Nadifloxacin	95.97854672	105.3161387	197.7919092	49.69666055
Imatinib	97.51970388	93.07267891	136.4327545	63.45189231
Moxifloxacin	113.2848862	118.0479756	0	106.2729845
Formoterol fumarate	129.0877743	118.43763	178.5337862	109.7632068
Rufloxacin	69.26948846	120.9340719	172.9002306	35.31572526
Pravastatin	124.228637	81.49659264	197.2409642	112.2780434
Rosiglitazone Hydrochloride	106.8158203	107.5056263	130.559057	98.06171216
Rivastigmine	84.62919025	137.5567832	197.8099406	106.2788603
Sildenafil	123.0455426	132.5152974	161.9672638	147.5880583
Acetylsalicylic acid	126.4361161	121.1543333	192.7058694	129.2469284
Hexachlorophene	0	101.8034454	0	0
	**10 μM Replicate 1**	**10 μM Replicate 2**	**100 μM Replicate 1**	**100 μM Replicate 2**
**SSMD values**	6.85 ± 3.47	3.85 ± 1.52	4.91 ± 0.39	5.15 ± 3.04
On-plate controls	DMSO/Glucose	DMSO/Glucose	DMSO/Fusidic acid	DMSO/Glucose

*Was performed as the first pilot screen and used DMSO, fusidic acid controls
